# Richer than Gold: the fungal biodiversity of Reserva Los Cedros, a threatened Andean cloud forest

**DOI:** 10.1186/s40529-023-00390-z

**Published:** 2023-07-06

**Authors:** R. Vandegrift, D. S. Newman, B. T. M. Dentinger, R. Batallas-Molina, N. Dueñas, J. Flores, P. Goyes, T. S. Jenkinson, J. McAlpine, D. Navas, T. Policha, D. C. Thomas, B. A. Roy

**Affiliations:** 1 Inst. of Ecology and Evolution, Department of Biology, University of Oregon, Eugene, OR 97402 USA; 2 Glorieta, NM USA; 3 Biology Department and Natural History Museum, University of Utah, Salt Lake City, Utah USA; 4 Herbario Nacional del Ecuador (QCNE), sección botánica del Instituto Nacional de Biodiversidad (INABIO), Avenida Río Coca E6-115 e Isla Fernandina, Sector Jipijapa, Quito, Ecuador; 5 Departamento de Investigación de Mycomaker, Quito, Ecuador; 6 Departamento de Investigación de Reino Fungi, Quito, Ecuador; 7 Microbiology Institute-Universidad San Francisco de Quito, Quito, Ecuador; 8 Department of Biological Sciences, California State University, East Bay, Hayward, CA USA; 9 Bayreuth Center of Ecology and Research, University of Bayreuth, Bayreuth, Bayern, DE Germany

**Keywords:** Anamorph-teleomorph connections, Ecuador, Conservation, Diversity, iNaturalist, Agaricales, Xylariales, Fungi, Ecology, Tropical

## Abstract

**Background:**

Globally, many undescribed fungal taxa reside in the hyperdiverse, yet undersampled, tropics. These species are under increasing threat from habitat destruction by expanding extractive industry, in addition to global climate change and other threats. Reserva Los Cedros is a primary cloud forest reserve of ~ 5256 ha, and is among the last unlogged watersheds on the western slope of the Ecuadorian Andes. No major fungal survey has been done there, presenting an opportunity to document fungi in primary forest in an underrepresented habitat and location. Above-ground surveys from 2008 to 2019 resulted in 1760 vouchered collections, cataloged and deposited at QCNE in Ecuador, mostly Agaricales sensu lato and Xylariales. We document diversity using a combination of ITS barcode sequencing and digital photography, and share the information via public repositories (GenBank & iNaturalist).

**Results:**

Preliminary identifications indicate the presence of at least 727 unique fungal species within the Reserve, representing 4 phyla, 17 classes, 40 orders, 101 families, and 229 genera. Two taxa at Los Cedros have recently been recommended to the IUCN Fungal Red List Initiative (*Thamnomyces chocöensis* Læssøe and “*Lactocollybia” aurantiaca* Singer), and we add occurrence data for two others already under consideration (*Hygrocybe aphylla* Læssøe & Boertm. and *Lamelloporus americanus* Ryvarden).

**Conclusions:**

Plants and animals are known to exhibit exceptionally high diversity and endemism in the Chocó bioregion, as the fungi do as well. Our collections contribute to understanding this important driver of biodiversity in the Neotropics, as well as illustrating the importance and utility of such data to conservation efforts.

**Resumen:**

*Antecedentes*: A nivel mundial muchos taxones fúngicos no descritos residen en los trópicos hiper diversos aunque continúan submuestreados. Estas especies están cada vez más amenazadas por la destrucción del hábitat debido a la expansión de la industria extractivista además del cambio climático global y otras amenazas. Los Cedros es una reserva de bosque nublado primario de ~ 5256 ha y se encuentra entre las últimas cuencas hidrográficas no explotadas en la vertiente occidental de los Andes ecuatorianos. Nunca antes se ha realizado un estudio de diversidad micológica en el sitio, lo que significa una oportunidad para documentar hongos en el bosque primario, en hábitat y ubicación subrepresentatadas. El presente estudio recopila información entre el 2008 y 2019 muestreando material sobre todos los sustratos, reportando 1760 colecciones catalogadas y depositadas en el Fungario del QCNE de Ecuador, en su mayoría Agaricales sensu lato y Xylariales; además se documenta la diversidad mediante secuenciación de códigos de barras ITS y fotografía digital, la información está disponible en repositorios públicos digitales (GenBank e iNaturalist). *Resultados:* La identificación preliminar indica la presencia de al menos 727 especies únicas de hongos dentro de la Reserva, que representan 4 filos, 17 clases, 40 órdenes, 101 familias y 229 géneros. Recientemente dos taxones en Los Cedros se recomendaron a la Iniciativa de Lista Roja de Hongos de la UICN (*Thamnomyces chocöensis* Læssøe y *“Lactocollybia” aurantiaca* Singer) y agregamos datos de presencia de otros dos que ya estaban bajo consideración (*Hygrocybe aphylla* Læssøe & Boertm. y *Lamelloporus americanus* Ryvarden). *Conclusiones:* Se sabe que plantas y animales exhiben una diversidad y endemismo excepcionalmente altos en la bioregión del Chocó y los hongos no son la excepción. Nuestras colecciones contribuyen a comprender este importante promotor de la biodiversidad en el Neotrópico además de ilustrar la importancia y utilidad de dichos datos para los esfuerzos de conservación.

**Supplementary Information:**

The online version contains supplementary material available at 10.1186/s40529-023-00390-z.

## Background

Global estimates for fungal diversity have ranged from 500,000 to 10 million over the course of the last century, the most recent estimate narrowing that range to 2.2–3.8 million (Hawksworth and Lücking 2017), of which only ~ 150,000 have been described to science (Lücking et al. 2021). With uncertainty surrounding the precise scope and extent of Earth’s fungal diversity, a consensus has emerged that the majority of this diversity, both known and unknown, resides in the tropics (Tedersoo et al. 2014; Hu et al. 2019; Hawksworth & Lücking 2017). Certain tropical regions have received ample mycological attention, while others remain dramatically underexplored and understudied (Piepenbring 2015). Of the latter, the Andes mountain range is among the most diverse and least mycologically documented places on the planet (Geml et al. 2014; Simijaca et al. 2022; Ryvarden, pers. comm.).

While localities possessing this combination of hyperbiodiversity and underdocumentation should already be considered research priorities, we contend that a third qualifier—conservation status—should be taken into equal consideration. In recognition of these combined attributes, we have conducted an array of short- and long-term diversity and ecology studies within the threatened Ecuadorian protected forest, Reserva Los Cedros (RLC), a 5256-hectare preserve of mostly primary, pre-montane to montane, moist, broadleaf forest (*i.e.*, “cloud forest”). Here, we synthesize more than a decade of collecting work at Los Cedros to provide the first major exploration of one of its least known characteristics: its fungal biodiversity. This work draws from our previously published studies (Policha 2014; Policha et al. 2016; Thomas et al. 2016; Nelson et al. 2020), as well as new collections, including many from previously unexplored parts of the Reserve.

### History of mycology in Ecuador

While uses of fungi among indigenous Andean-Amazonian peoples go back many thousands of years (Fidalgo and Prance 1976; Davis and Yost 1983; Prance 1984; Zent et al. 2004; Gamboa-Trujillo 2005; Zent 2008), the contemporary field of mycology in Ecuador has a relatively brief history. The most notable early contributor was Nils Gustav de Lagerheim (1889–1895), a Swedish botanist and plant pathologist, and a founder of modern Ecuadorian mycology, who often published in collaboration with Narcisse Théophile Patoulliard in Paris (Læssøe and Petersen 2011a). The next sizable contribution was provided by Hans Sydow, who visited Ecuador in 1937, and collected primarily microfungi (Sydow et al. 1939; Petrak and Others 1948). Though much of Sydow’s material was lost during the second world war, more than 180 fungal species described from Ecuador are based on his types, and at least 17 Ecuadorian taxa are named in his honor (Læssøe and Petersen 2011a).

The 1970s were a period of renewed mycological investigation in Ecuador, attracting the attention of Rolf Singer (1973), Harry C. Evans (1973–1975), and Kent Dumont (1975), focusing (primarily) on the “higher” basidiomycetes, entomo- and phyto-pathogenic fungi, and inoperculate discomycetes, respectively. Dumont alone amassed some 2,300 collections of Ecuadorian fungi, deposited at NY (Læssøe and Petersen 2011a). In 1993, the British Mycological Society organized an internationally-attended expedition to Ecuador (Lodge and Cantrell 1995; Lodge 1996; Lunt and Hedger 1996), attracting some 30 participants and generating upwards of 1600 collections, duplicates of which are housed at PUCE.

At the start of the twenty-first century, Danish mycologists Thomas Læssøe and Jens Petersen set out to create what would ultimately become one of the most significant contributions not only to Ecuadorian mycology, but to the study of tropical American fungi as a whole. Over the course of several field expeditions (2001–2004), Læssøe and Petersen generated both well-documented collections and high-quality, in situ, color photography for roughly 1200 species of Ecuadorian fungi. They also assembled the first comprehensive bibliography of Ecuadorian mycological literature, out of which was born the first Ecuadorian national checklist of fungi numbering 3,766 taxa (Læssøe and Petersen 2008). While certainly a gross underestimate of Ecuador’s actual fungal diversity, the list represents an important baseline for further study, particularly in combination with data from the Ecuadorian National Herbarium (QCNE) (Batallas-Molina et al. 2020). The sum of these collection and curatorial efforts went on to form the Ecuador section of the pair’s pioneering *MycoKey* website, a resource without equal in the identification and appreciation of Andean-Amazonian funga. From 2004 to 2011, *MycoKey Ecuador* would serve as the only open-aceess, large-scale collection of high-quality, color photographs of macrofungi from the South American continent (Læssøe and Petersen 2011b).

More recently there has been an acceleration of mycological research in Ecuador, and a notable transition to studies undertaken by Ecuadorian researchers. These include works on wood decay fungi (Ullah et al. 2001; Suárez-Duque 2004; Gehring and Batalles 2020), mycorrhizal fungi (Kottke et al. 2010, 2013; Novotná et al. 2018), and ethnomycology (Gamboa-Trujillo 2005; Gamboa-Trujillo et al. 2014), along with various taxonomic and ecological studies (*e.g.,* (Barili et al. 2017a, b, c, 2018; Flores et al. 2018; Guevara et al. 2018; Toapanta-Alban et al. 2021, 2022).

Our own research timeline began in January of 2008. Five more expeditions followed over the course of the next ten years (2010, 2011, 2012, 2014, 2018), resulting in a variety of focused publications addressing specific research questions (Dentinger and Roy 2010; Policha and Roy 2012; Policha et al. 2016; Thomas et al. 2016; Nelson et al. 2020). To date, no prior publication from our work at Los Cedros has sought to comprehensively address the sum of our fungal collections from the site, or explore their implications.

### Threats to Los Cedros

In contrast to many of our mycological predecessors of the last two centuries—who would have found it relatively easy to locate large, dense swaths of primary rainforest in which to collect data over a long period—the biodiversity researcher of the twenty-first century is increasingly required to dedicate at least as many resources to protecting their habitats of interest as they do to simply studying them, lest there be no habitats left to study. This has contributed to an evolving paradigm shift in the planning, execution, and conceptualization of biodiversity research, and the roles and responsibilities of biodiversity researchers (Zedler 1997; Franco 2013; Darwall et al. 2018). In few places has this been truer than the perennially-imperiled but fiercely-defended cloud forests of Reserva Los Cedros (Torre 2012; Vandegrift et al. 2017; Roy et al. 2018; Guayasamin et al. 2021, 2022).

Since its founding in 1988, Reserva Los Cedros has been under near-constant threat, despite in 1994 being formally designated a *bosque protector*, a class of protected forest under Ecuadorian law. Historically, deforestation for conversion to pasture, colonization, and hunting have been the major threats to the forest, but recent years have seen an increased threat from large-scale extractive industry, not only at Los Cedros but throughout the Ecuadorian Andes (Vandegrift et al. 2017; Roy et al. 2018; Acosta et al. 2020).

In 2016, mining concessions covering 68% of the land area of Los Cedros were granted to a Canadian company in a joint venture with the Ecuadorian national mining company (ENAMI). This set up a years-long legal battle between the protected forest and the mining companies seeking to exploit it, with implications not only for protected forests in Ecuador, but for the global movement towards granting rights directly to nature (Guayasamin et al. 2021). The case worked its way to Ecuador’s Constitutional Court, the highest in the nation. In December 2021, the Constitutional Court chose to uphold the landmark Rights of Nature provisions in Ecuador’s constitution (Article 71–74), safeguarding Reserva Los Cedros from the threat of mining (Jimenez, 2021). However, despite mining within the protective forest being prohibited outright in the decision, the mining concessions covering the Reserve remain active in the official registry of the regulatory entity (ARCERNNR, 2023), and the mining companies continue to be active in the region, though not within the limits of the Reserve.

Here, we provide a preliminary account of the macrofungal diversity within an ecosystem considered to be a conservation priority, recognizing that the first step toward bringing conservation efforts for funga into parity with those of flora and fauna is documentation.

## Methods

### Study site

**Fig. 1:**
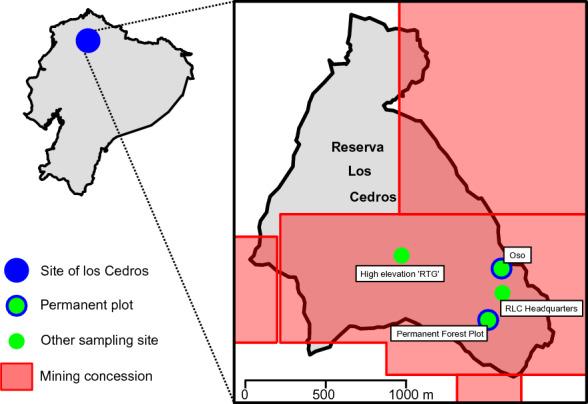
Map showing the location of Reserva Los Cedros within Ecuador; inset shows the primary sampling locations, and the overlay (red) shows the extent of mining concessions affecting the Reserve (see above, Threats to Los Cedros)

Bosque Protector Reserva Los Cedros is a 5256 hectare preserve consisting of mostly (> 84%) primary cloud forest ranging in elevation between 1000 and 2700 m (Fig. 1). Los Cedros is at the southern boundary of the Chocó bioregion in the Toisin Range, which extends west from the western slopes of the Andes mountains in northwest Ecuador. Rainfall measurements at the Field Station, at 1395 m, indicate that 2903 ± 186 mm of rain falls annually (Jose DeCoux, pers. comm.) and far more rain falls on the ridges. Reserva Los Cedros hosts an exceptionally rich diversity of plants and animals (Roy et al. 2018; Wilson and Rhemtulla 2018; Ramirez Perez and Hausdorf 2022; Mariscal et al. 2022), and as indicated by the findings of the present work, is also home to a comparable degree of fungal diversity.

### Collecting methods

Through a combination of plot/transect sampling (see *Ecological Collections* below), opportunistic collecting, and focused sampling of particular taxonomic groups (*e.g.*, Xylariaceae), we have, over the course of a 11-year period spanning six separate collecting trips, generated over 1700 fungal collections from along the 1700 m altitudinal gradient of Reserva Los Cedros. Primary sampling locations were near to the research station, within or near the two permanent diversity plots, and at the high-elevation ‘Richer Than Gold’ expedition site, which was sampled in late 2018.

While methods varied somewhat over the course of sampling, collection protocols generally adhered to the following principles: assigning of unique collection numbers; annotation and photo documentation of fresh specimens; geotagging of collections; tissue sampling for use in molecular work; preservation via desiccation; and duplication (when not singletons) for dual deposit at the Herbario Nacional del Ecuador (QCNE) del Instituto Nacional de Biodiversidad (INABIO) and one or more secondary research institutions (OSC and K, primarily). The specimens collected and deposited at QCNE from the period 2008–2018 correspond to the cataloging numbers QCNE201900-201999; QCNE242449-242548, QCNE244173-244890; QCNE246391-246500; QCNE247390-247547.

All herbarium codes follow Index Herbariorum (2018). Specimen images, notes and metadata were entered into individual observations on iNaturalist, where they will be linked with their respective voucher records on MycoPortal, as well as any associated accession numbers for sequence data uploaded to GenBank. Desiccation was achieved through the use of silica gel, a portable dehydrator at or below 43 °C, or both.

Beginning in 2014 (collections RLC1173–RLC1854), we began selectively employing a photographic technique known as “focus stacking”, via the computer program Zerene Stacker (v. 1.00-1.04; Littlefield, 2014–2023), to address the significantly reduced depth of field which accompanies macro photography, particularly at higher magnifications.. Photographs generated during this period were also subject to color calibration using an X-Rite ColorChecker Passport and a display colorimeter (ColorMunki Display & Eizo EX4).

### Ecological collections

To compare communities in different parts of the Reserve, we set up plots in which the collecting was done at the same time of year (January) and in as short a time period as possible (3–4 days) per plot. In 2010, two plots of parallel transects were established about 1 km apart within the Reserve in two different habitats: ridgetop at 1666 m and riverbottom at 1322 m. The ‘Oso Ridge Fungus Plot’ consisted of two 300 m long transects along the Oso ridge, separated by 10 m, while the ‘Permanent Forest Plot’ was within the 1 ha ‘Permanent Tree Diversity Plot’ established by Peck in 2005 (Peck et al. 2008; Mariscal et al. 2022) on the banks of the Los Cedros River, and consisted of ten 55 m and one 45 m long transects. The different arrangements of the transects at the two sites was necessary due to the restricted area of traversable terrain. In all cases the sampling points were 5 m apart along the transects and 10 m between transects. The sampled points consisted of a circle with a radius of 1.2 m around each point (= 4.42 m^2^) for a combined total of 542.4 m^2^ per plot. Within each sampled point we looked for macrofungi on all surfaces, including standing or dead wood up to 1.5 m in height; equal sampling intensity was applied to all plots.

All fungi within each sample area were recorded for morphospecies present, and the number of fruiting bodies was counted. Each new morphospecies encountered within the plots was vouchered for future identification. In practice, this meant we counted but did not always collect the myriad of small, white-spored, litter-decomposing, marasmioid and mycenoid agarics. Surveys were done in mid January in 2010, 2011 and 2012, but due to how the data were recorded in each year, the data could not be combined for all analyses. In 2010, under the direction of B. Roy, we counted and collected representatives of both ascomycetes and basidiomycetes; in 2011, under B. Dentinger, the focus was basidiomycetes; in 2012, under R. Vandegrift, the focus was the genus *Xylaria* Hill ex Schrank and only at the lower (Brazilargo) plot (Policha 2014; Policha et al. 2016; Thomas et al. 2016).

### Statistical analysis

Species richness in the plots was estimated using Chao2 and Jacknife1 estimators (Burnham and Overton 1978; Chao 1984; Colwell and Coddington 1994). Collections from ecological sampling were used to compare communities between lower elevation and higher elevation sites; data were subsetted from the full dataset and converted into site-by-species matrixes, analyzed using both incidence (presence-absence) and abundance (number of fruiting bodies observed within the plot at the time of collection; note that the voucher collection may have been a subset of all fruiting bodies present). Community structure was analyzed using Jaccard (for incidence) or Bray–Curtis (for abundance) distances, visualized using non-metric multidimensional scaling (NMDS) and differences were assessed with permutational multivariate analysis of variance (PerMANOVA).

Data were analyzed using R Statistical Software (v3.1.0, R Core Team, 2014), including the *vegan* package (Oksanen et al. 2013). We also utilized the *reshape* and *dplyr* packages (Wickham 2007, 2009; Wickham et al. 2019) for data manipulation, and the *ggplot2* package (Wickham 2011) for visualization. All scripts, data tables, and raw data are available via an open FigShare repository (Vandegrift et al. 2023). Edited sequences have been uploaded to GenBank (accession numbers provided in Additional file 2).

### Sanger sequencing

DNA was extracted either by impregnation into Whatman FTA plantcards (Dentinger et al. 2010) or by suspension of dried material in an extraction buffer. We primarily sequenced the ITS1 and ITS2 regions using the primers ITS1F and ITS4 (White et al. 1990). For a subset of ± 100 Xylariaceae we added partial LSU (the ribosomal large subunit gene) by using the primers ITS1F and LR3 (Vilgalys and Hester 1990). For extraction and sequencing we used the protocol of Dentinger et al. (2010) when the DNA was on Whatman FTA cards, and of Thomas et al. (2016) otherwise, with the exception of RLC1-155, which were sequenced at BOLD (BarCode of Life Data Systems) in Guelph, Canada, and a subset of Xylariaceae, which were sequenced as test subjects by the North American Mycoflora Project (now FunDiS) in the Aime Lab (Purdue University, West Lafayette, Indiana). For sequence editing we used Geneious Prime (v2022.2, Dotmatics, Boston, MA).

### Identification

We have been conservative with our determinations as we were working in an understudied tropical area with many fungal groups for which we had no specialized expertise. For these reasons, we made frequent use of open nomenclature qualifiers (Sigovini et al. 2016) to indicate uncertainty. We use *confer* (cf.) to indicate that the collection in question should be compared to that taxon; the determination will likely be confirmed when examined by a specialist or compared to authentic reference material. We use *affinis* (aff.) to indicate that the collection has some affinity with the name applied, but differs in some potentially significant way; the name applied is the best determination we are able to make, and the collection is likely to be a closely related, but distinct, taxon. We use the qualifier sensu lato (s.l.) to indicate that a taxon name should be applied in the broad sense; ‘group’ to indicate that a taxon belongs to a group of similar, difficult to distinguish taxa epitomized by the name used; and, similarly, we use ‘complex’ to indicate that a taxon is part of a monophyletic grouping of difficult to distinguish taxa. Where possible, we follow previous conventions in the literature for the use of these qualifiers.

For about half the specimens, ITS sequences were used to aid in identification. We have translated the open nomenclature concepts into sequence similarity thresholds: we use *confer* at greater than 98% pairwise identities with reliable reference sequences, indicating that the determination will likely be confirmed when examined by a specialist or compared to authentic reference material; we use *affinis* at greater dissimilarity, 96–98% pairwise identities (or occasionally < 96% when also morphologically supported), to indicate that the best determination possible from reference sequences is likely a closely related taxon.

All sequences were initially compared to the UNITE (Abarenkov et al. 2010; Kõljalg et al. 2013; Nilsson et al. 2019) and the GenBank (Clark et al. 2016; Benson et al. 2017) *nr* databases using BLAST. We followed up by using BLAST distance trees to examine putative relationships among matches, and top sequence hits were examined in more detail, including but not limited to location of any publications utilizing those sequences and macromorphological comparison of our collections with images or other reference data (e.g., distribution, phylogenies). Comparison to primary literature is essential since GenBank does not permit non-author annotation (Bidartondo et al. 2008) and many fungal sequences are misidentified (Hofstetter et al. 2019). Current nomenclature was determined using Index Fungorum and Mycobank, except where contradicted by Jaklitsch et al. (2016) or *agaric.us* (Kalichman et al. 2020), which we gave priority for determining for the most current familial placements of genera of ascomycetes and agarics, respectively.

## Results

### Diversity checklists

**Fig. 2:**
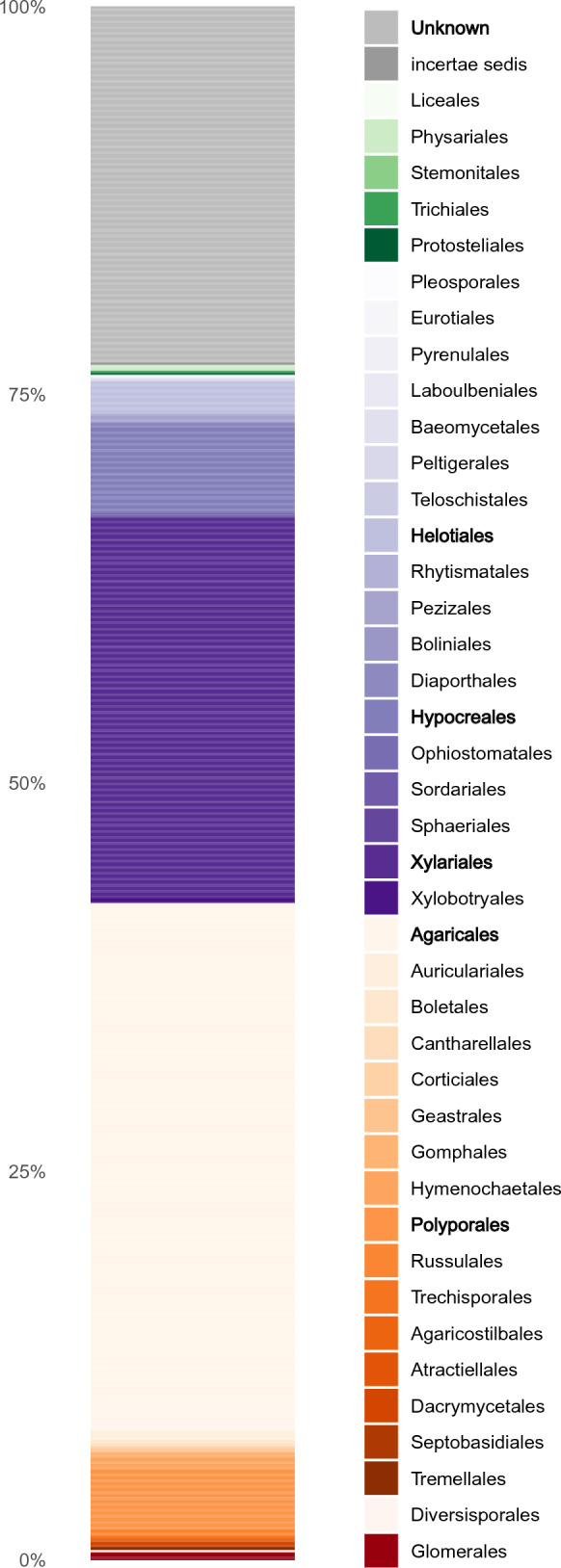
Relative number of collections, by assigned order; taxa representing relative abundances greater than 2.5% of all collections are listed in boldfaced, and color groups are used to differentiate phylum. There remain 106 collections for which an order-level determination has not yet been made (n = 1760 collections)

**Fig. 3:**
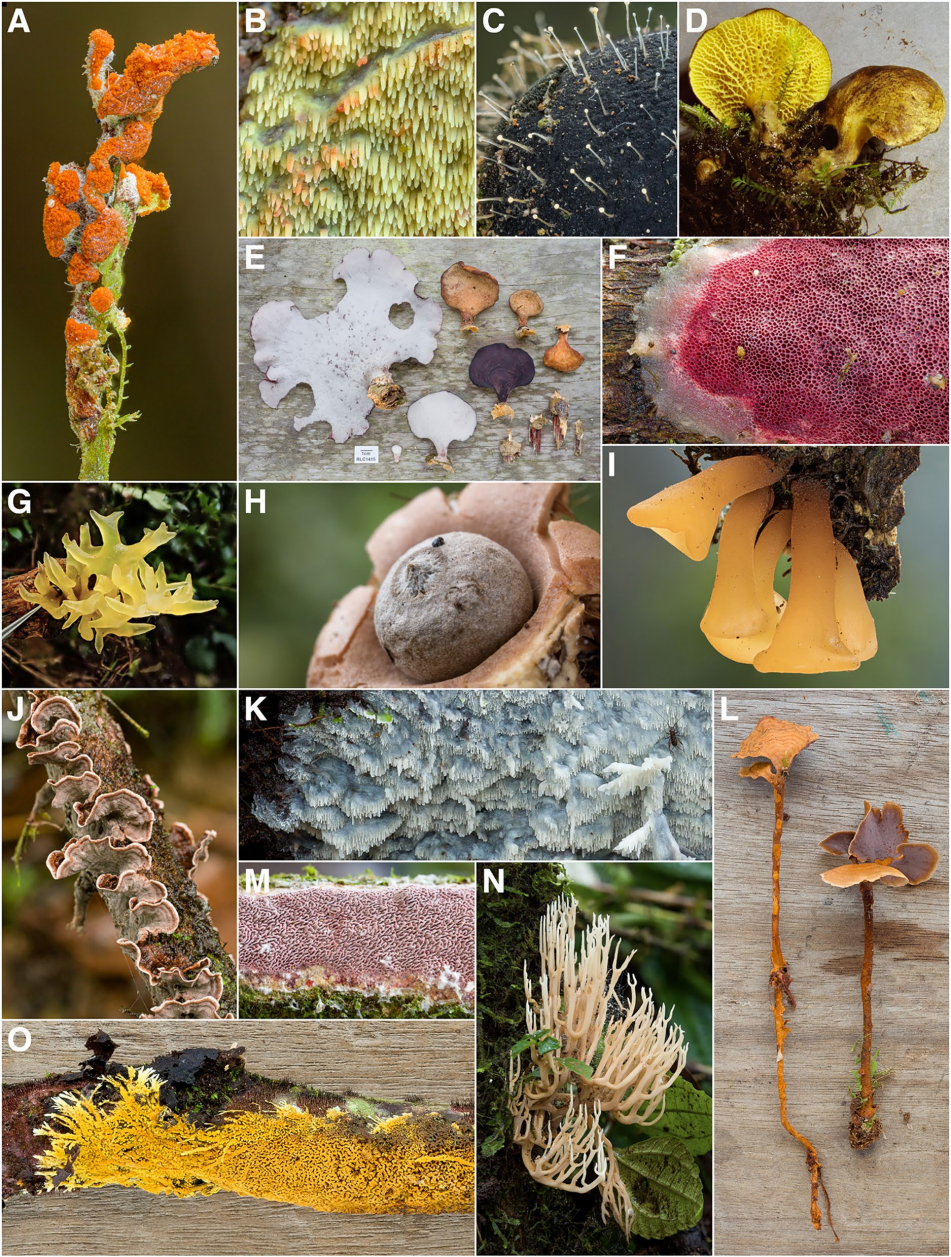
Some Diversity of Basidiomycota (excluding Agaricales) from Los Cedros. **A** *Botryobasidium sp.* [RLC1697] B *Geesterania cf. davidii* [RLC1264] **C** *Chionosphaera (*= *Fibulostilbum) phylaciicola* [RLC1611] **D** *Boletinellus exiguus* [RLC644] **E** *Polyporus iathinus* [RLC1415] **F** *indet.* Polyporales (*cf. Gloeoporus* / *cf. Skeletocutis*) [RLC1614] **G** *cf. Calocera* [RLC1824] **H** *Geastrum sp.* [RLC1514] **I** *cf. Dacryopinax* [RLC1612] **J** *Septobasidium sp. nov.* [RLC1602] **K** *Irpex rosettiformis* [RLC1177] **L** *Hymenochaete cf. damicornis* [RLC1511] **M** *Fuscoporia contigua* [RLC1233] **N** *Ramaria sp.* [RLC1263] **O** *cf. Lindtneria* [RLC1348]

**Fig. 4:**
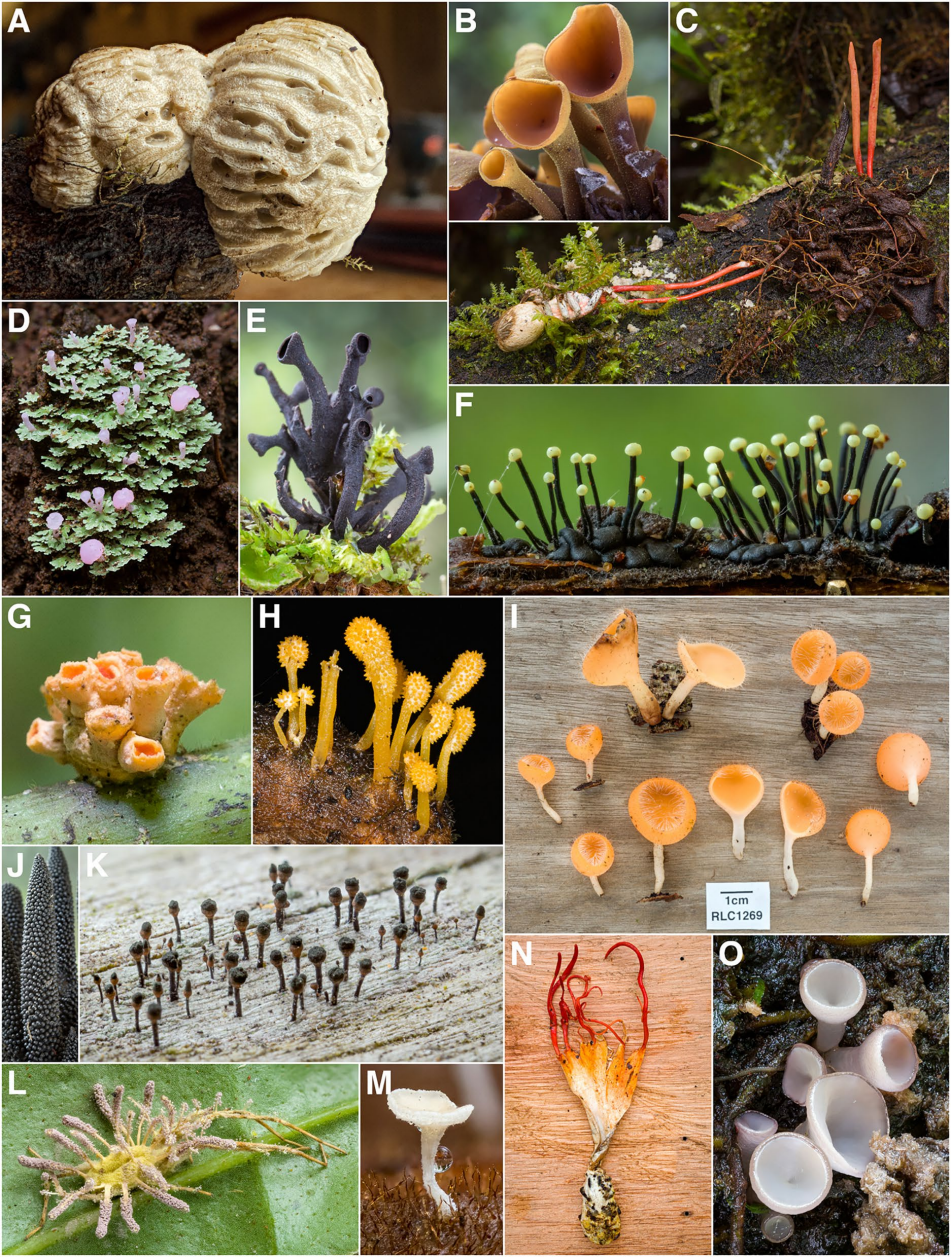
Some Diversity of Ascomycota (excluding Xylariales) from Los Cedros. **A** *Nectriopsis tremellicola* parasitizing a *Crepidotus sp.* [RLC1832] **B** *“Encoelia” heteromera* [RLC1380] **C** *Cordyceps pruinosa* group on indet. spider [RLC1718] **D** *Phyllobaeis sp.* [RLC1320] **E** Cordieritidaceae [RLC1466] **F** *Stromatographium stromaticum* (= *Fluviostroma wrightii*) [RLC1318] **G** *Moelleriella turbinata* [RLC1323] **H** *Cordyceps tenuipes* on lepidopteran pupa [RLC1687] **I** *Cookeina tricholoma* [RLC1269] **J** *Xylobotryum portentosum* [RLC1339] **K** Caliciaceae [RLC1211] **L** *Gibellula sp.* on indet. spider (collected in forest canopy ~ 75 m above forest floor) [RLC1799] **M** Lachnaceae [RLC1723] **N** *Cordyceps nidus* complex on trap door spider (Ctenzidae) [RLC1613] **O** *Neobulgaria sp.* [RLC1312]

During the course of this study, 1760 vouchered collections of fungi were made. Our findings indicate the presence of at least 727 unique species of macrofungi within Reserva Los Cedros, representing 229 genera in 101 families, 40 orders, 17 classes, and 4 phyla (Fig. 2). The vast majority of fungi collected were members of the phyla Basidiomycota (Fig. 3) and Ascomycota (Fig. 4). We provide two checklists to organize this information: a taxonomic list, structured hierarchically ( Additional file 1), and a collections list, structured by individual collection with full collection information, taxonomic classification, and associated accession number (herbarium and GenBank) provided for each specimen ( Additional file 2).

**Fig. 5:**
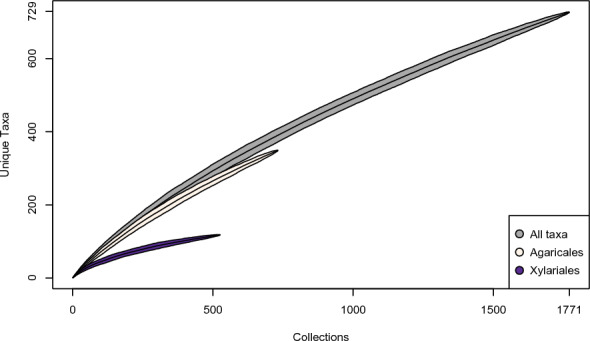
Collector’s curve, showing number of unique taxa recovered by all collections and the two most frequently encountered orders, Agaricales and Xylariales

**Fig. 6:**
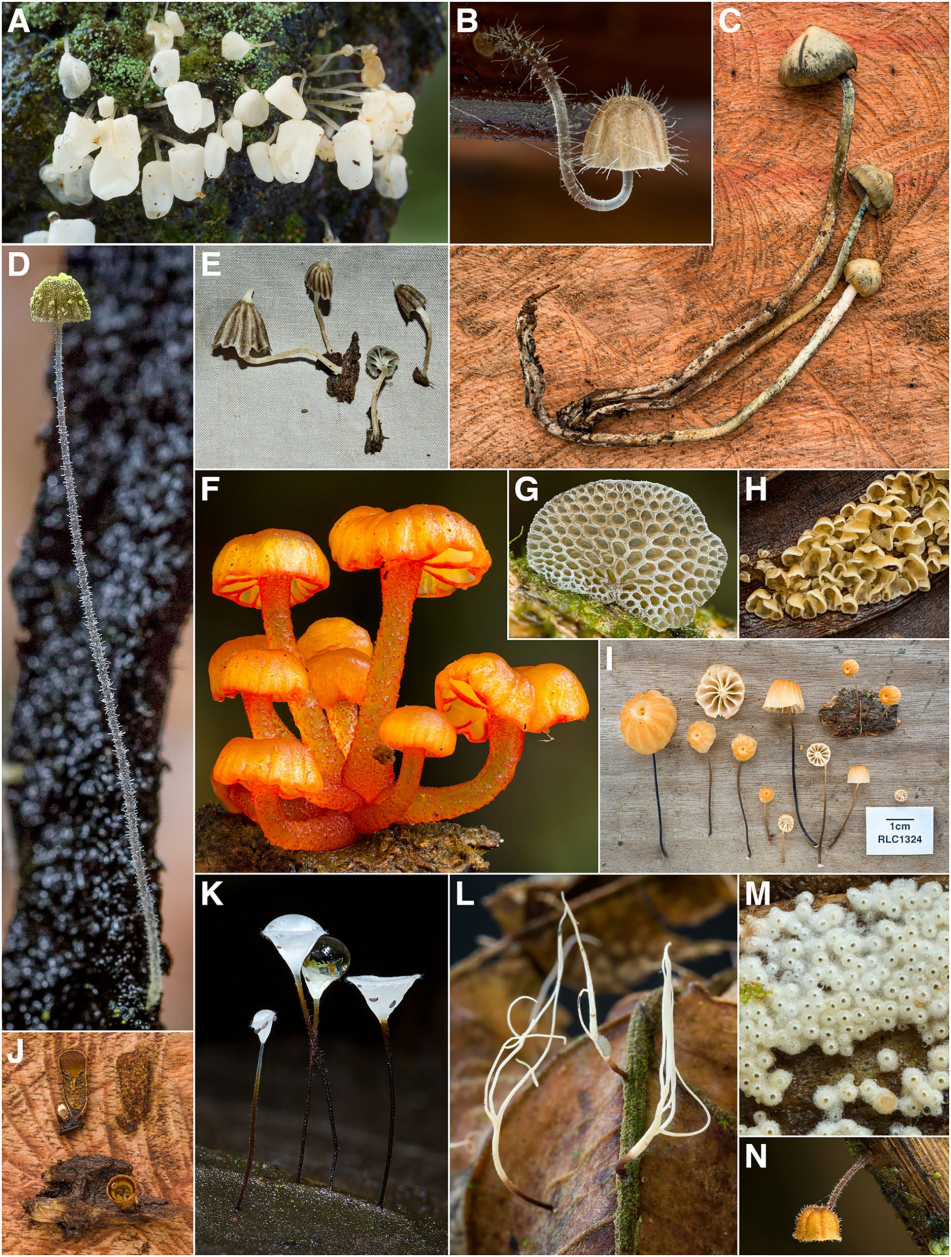
Some Diversity of Agaricales from Los Cedros. **A** *Physalacria sp. nov.* [RLC1310] **B** *Mycena sect. Longisetae* [RLC1662] **C** *Psilocybe zapotecorum* [RLC1610] **D** *Mycena chloroxantha* [RLC1293] **E** *Hydropus sp.* [RLC128.1] **F** *indet.* Mycenaceae s.l. [RLC1784] **G** *Favolaschia sp.* [RLC1775] **H** *Calathella columbiana* [RLC1686] **I** *Marasmius sect. Marasmius* [RLC1324] **J** *Cyathus sp.* [RLC1679] **K** *Gloiocephala sp.* [RLC1289] **L** *Pterulicium sp.* [RLC1268] **M** *indet.* Cyphellaceae s.l. [RLC1772] **N** *indet.* Physalacriaceae (*cf. Rhizomarasmius / cf. Gloiocephala*) [RLC1720]

**Fig. 7:**
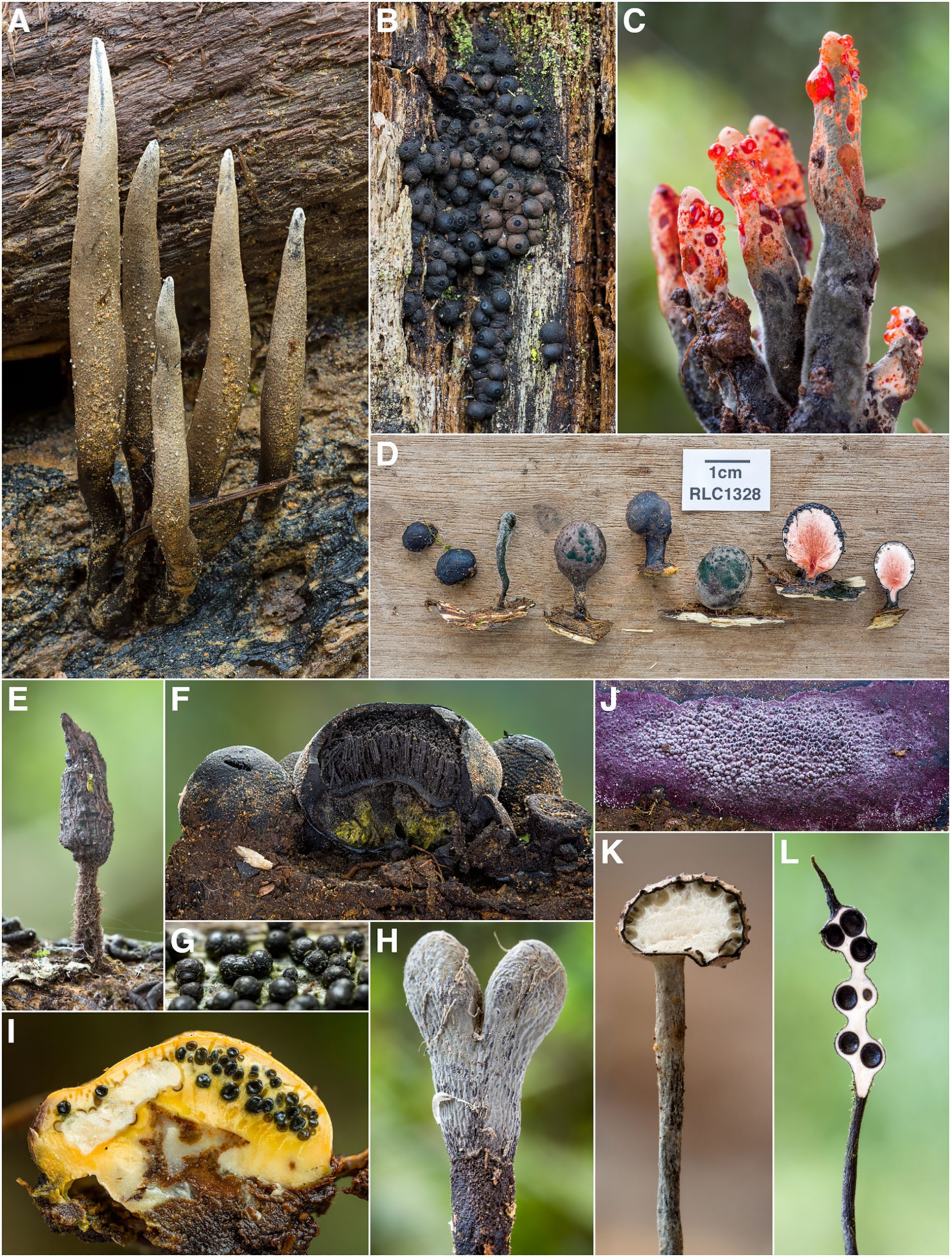
Some Diversity of Xylariales from Los Cedros. **A** *Xylaria telfairii* [RLC1203] **B** *Annulohypoxylon sp.* [RLC1228] **C** anamorph of *Xylaria globosa* with characteristic red exudates [RLC1344] **D** *Xylaria tuberoides* [RLC1328] **E** *Xylaria apiculata* [RLC1469] **F** section of *Phylacia poculiformis* [RLC1601] **G** *Rosellinia sp.* [RLC1173] **H** *Xylaria schweinitzii* (anamorph/immature) [RLC1335] **I** *cf. Thuemenella* [RLC1827] **J** *Hypoxylon sp.* [RLC1176] **K** section of *Xylaria clusiae* [RLC1480] **L** section of *Xylaria melaneura* group [RLC1378]; this collection was previously identified as *X. tucumanensis* at the time this photo appeared on the cover of *Biotropica* 48(3) accompanying Thomas et al. (2016)

These figures only begin to approach the true magnitude of the fungal diversity within the Reserve (Fig. 5). The Chao2 richness estimator predicts at least twice as many taxa present, 1671 total species; the Jackknife 1 estimator is somewhat more conservative, estimating 1,205 total species. Both are almost certainly underestimates, given existing sampling biases. Such richness estimates are susceptible to influence from sampling biases introduced by project participants, such as when collections were made in the service of ecological projects (Policha et al. 2016; Thomas et al. 2016; Policha et al. 2019). These biases have a clear effect on the taxonomic coverage of the fungi collected (Fig. 2), specifically leading to overrepresentation of the Agaricales (Fig. 6) and the Xylariales (Fig. 7) within our dataset. An examination of these well-sampled orders reveals a smaller gap in sampled and estimated diversity, particularly in the Xylariales, within which the genus *Xylaria* is especially well sampled (Fig. 5). In the Xylariales, we have recorded 118 unique taxa, or 61% of the Chao2 richness estimator prediction of 193 total species; in contrast, the ratio of sampled to estimated species for the total set of collections predicts only 43.5% complete sampling. Interestingly, despite the over-representation of Agaricales in our collections, the ratio of sampled to predicted richness remains similar to the total dataset (42.9%).

### Ecological sampling

**Fig. 8:**
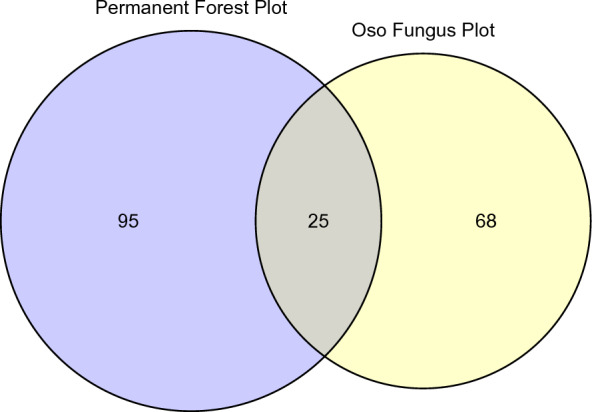
Shared species between the riverbottom (Permanent Forest Plot) and ridgetop (Oso Fungus Plot) sampling in 2010

**Fig. 9:**
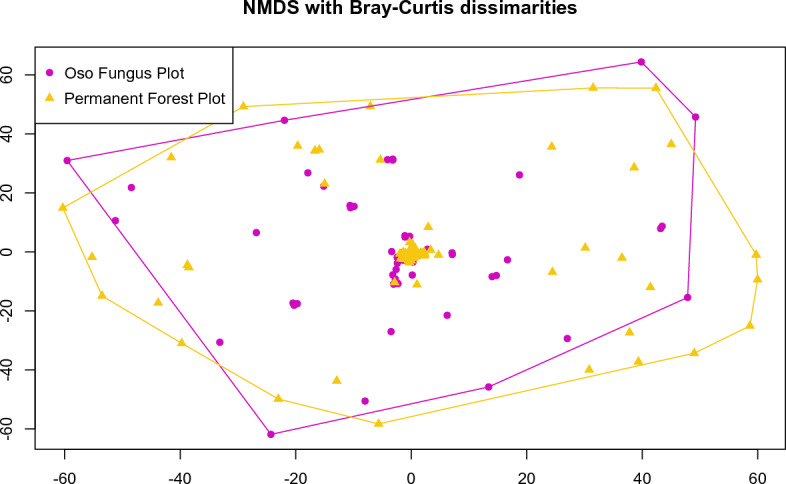
NMDS ordination on Bray–Curtis dissimilarities; there is no significant difference between the riverbottom (Permanent Forest Plot) and ridgetop (Oso Fungus Plot) sites

Macrofungal communities sampled systematically in 2010 from the two permanent forest plots at Los Cedros, representing lower elevation riverbottom (Permanent Forest Plot) and higher elevation ridgetop (Oso Fungus Plot) habitats, resulted in 354 vouchered collections representing count data taken within each point of each plot (Additional file 3). These collections were used to compare the two communities at the two plots. Despite only sharing 13% of taxa (25 taxa; Fig. 8), we observed no statistically significant differences between fungal communities at lower and higher elevation sites (Fig. 9; PerMANOVA (abundance with Bray–Curtis distances): F_1, 190_ = 1.43, R^2^ = 0.007, *P* = 0.083; PerMANOVA (incidence with Jaccard distances): F1, 190 = 1.41, R2 = 0.007, P = 0.053). Furthermore, of the shared taxa, only nine could be identified to the level of species, meaning it is likely that the degree of shared taxa is even less than reported here (see Additional file 3 for differential abundance data).

## Discussion

### Diversity and ecology

This investigation of fungi at Reserva Los Cedros contributes to the long history of mycology in Ecuador, providing some of the most comprehensive documentation of fungal diversity within montane cloud forests anywhere in the world (Læssøe and Petersen 2008, 2011b; Lodge et al. 2008; Gómez-Hernández and Williams-Linera 2011; Geml et al. 2014; Del Olmo-Ruiz et al. 2017; Gehring and Batalles 2020; Haelewaters et al. 2021). We have contributed 905 ITS sequences connected to vouchered and well-documented specimens to GenBank, of which more than 10% have no close matches (> 90% pairwise identities) within the GenBank *nr* database. That richness estimates for the most frequently collected orders—Agaricales and Xylariales—are still far from saturation suggests that even with targeted, multi-year collecting of single fungal groupings, novel taxa may be expected to be recovered for many years to come within forests of this type. Many putatively undescribed taxa are documented for the first time here, including new species of *Chalciporus* (Boletaceae), *Psilocybe* (Hymenogastraceae s.l.), *Ionomidotis* (Cordieritidaceae), *Kretzschmaria* and *Xylaria* (Xylariaceae).

The comparative ecological sampling in 2010, which included broad taxonomic coverage sampled intensively and systematically between two different habitats, seems at first to have generated contradictory results: the riverbottom habitat and the ridgetop habitat were found to share only 25 species, out of a total pool of 188 individual taxa (Fig. 8); our multivariate statistical analysis, however, failed to recover a significant difference between the communities (change in beta-diversity), as would be expected (Fig. 9). The seeming discrepancy between these results is likely explained by undersampling relative to the high diversity present within the sites, with most taxa being sampled only once or twice in this subset of our data; as such, multivariate statistical approaches to community analysis are severely underpowered to detect differences, even when present, as is likely the case here. This is a stark demonstration of the degree of sampling effort necessary to characterize fungal communities in tropical cloud forests well enough to test for changes in beta-diversity.

### “Simulated Access” & parataxonomy

Historically, the documentation of fungal collections has been dominated by written descriptions, occasionally supplemented by illustration or photography. Such descriptions are highly technical and often taxon-specific, requiring a working knowledge of diagnostic features and the terminology used to describe them for specific taxonomic groups. This presents a problem for researchers engaged in more broadly-focused field work, as in the case of our collecting efforts.

These same constraints are partly responsible for the preponderance of taxonomic descriptions and decisions based on dried material; a practice whose shortcomings are perhaps best exemplified by the revelations found in Hans-Otto Baral’s system of “vital taxonomy” (Baral 1992). While Baral’s findings pertain chiefly to certain microscopic structures found in the discomycetes, they stand as a testament to the general ephemerality and elusivity of so many fungal features, which may remain unknown even to their specialists for centuries. In recognition of this, combined with our team’s limited technical capacity to both recognize and describe a given fungal group’s most nuanced characters, we have used high quality, color-calibrated, digital photography (and in select instances, videography) to provide future specialists with a degree of “simulated access” to fresh specimens, from which they would otherwise be temporally and spatially separated. Characters uncapturable by photography (e.g.: odor, taste, texture/consistency) are recorded in the traditional written form, as the lexicon of descriptive terms for these qualities is more or less the same from one macrofungal group to another. The specialist, being the more qualified party, may then articulate their own written descriptions. This practice alleviates the need for plurality of taxonomic proficiency on the part of the collector(s), increases visual objectivity over linguistic subjectivity, and records details whose diagnostic value may not be comprehended for years to come.

High-quality photodocumentation presents an additional benefit in the form of providing taxonomic machine learning algorithms with feature-rich source imagery (Joly et al. 2014; Wäldchen and Mäder 2018), whether used in tandem with other parameters or in isolation. These methods are already being experimentally employed across biological taxonomy (Sun et al. 2017; Bambil et al. 2020; Mahmudul Hassan and Kumar Maji 2021; Høye et al. 2021), including fungi (Picek et al. 2022; Bartlett et al. 2022), and are poised to offer insights otherwise unattainable by existing taxonomic expertise. It is important to regard such innovations as individual components of the complete taxonomic toolkit, as overreliance on new and groundbreaking tools can have demonstrably deleterious effects, as has occurred with DNA sequencing (Bidartondo et al. 2008; Hofstetter et al. 2019).

We consider this approach to be an extension of the parataxonomic model first described by entomologist Daniel Janzen (Janzen 1991). Janzen drew attention to the magnitude of the planet’s still-undiscovered biodiversity, coupled with contemporary rates of taxonomic description of novel taxa, and inferred that, barring some exponential change in either variable, it would be several thousand years before humanity would achieve total taxonomic documentation of all life on Earth. To address this problem, he proposed a division of taxonomic labor. The tasks for which specialized taxonomic training is not required (*e.g.*, travel arrangements, permit acquisition, specimen documentation, preservation, deposit/duplication, etc.) would fall to a new, assistive class of biodiversity researcher: the “parataxonomist”. This would, in turn, free up precious time and resources for the “alphataxonomist” to focus on those tasks which their highly specialized expertise renders them uniquely qualified to address (e.g., precise identifications, descriptions of novel taxa, nomenclatural considerations, inferring evolutionary relationships, identifying target taxa for additional sequencing, etc.).

We have found great value in the parataxonomic model for its ability to facilitate existing research relationships (DSN first entered the project partly as a parataxonomist), as well as for its ability to function in a prefigurative sense, laying the groundwork for future collaborations. By generating a great variety of interesting, high-quality collections—specifically selected for their known or perceived taxonomic significance—we hope to appeal to the research interests of a wide range of alphataxonomists: a kind of “taxonomic brochure” for the fungi of Los Cedros, the Chocó bioregion, and the Andes as a whole. Such engagement is expected to multiply the total biodiversity research output of the project, hastening the comprehension of Reserva Los Cedros’ megadiverse funga, and in turn strengthening the Reserve’s conservation status.

Scaling, systematizing and tailoring these roles to meet Ecuador’s unique circumstances could bring about scientific, economic and conservational achievements in Ecuador on par with, if not exceeding, those experienced by Costa Rica during its parataxonomic heyday (Kazmier 2017).

### Notable collections from Los Cedros

In addition to corroborating estimates of fungal hyperdiversity in Ecuador’s Chocó bioregion, our research has also resulted in a wide variety of novel taxonomic insights, including the discovery of several putatively new taxa, from across many orders of macrofungi. In keeping with the concepts laid out in the previous section, the following synopses are presented as an abbreviated, representative sampling of collections which are known or suspected to deserve further alphataxonomic inquiry (see Additional file 2 for corresponding accession data). The findings discussed below should therefore be considered preliminary.

**Fig. 10:**
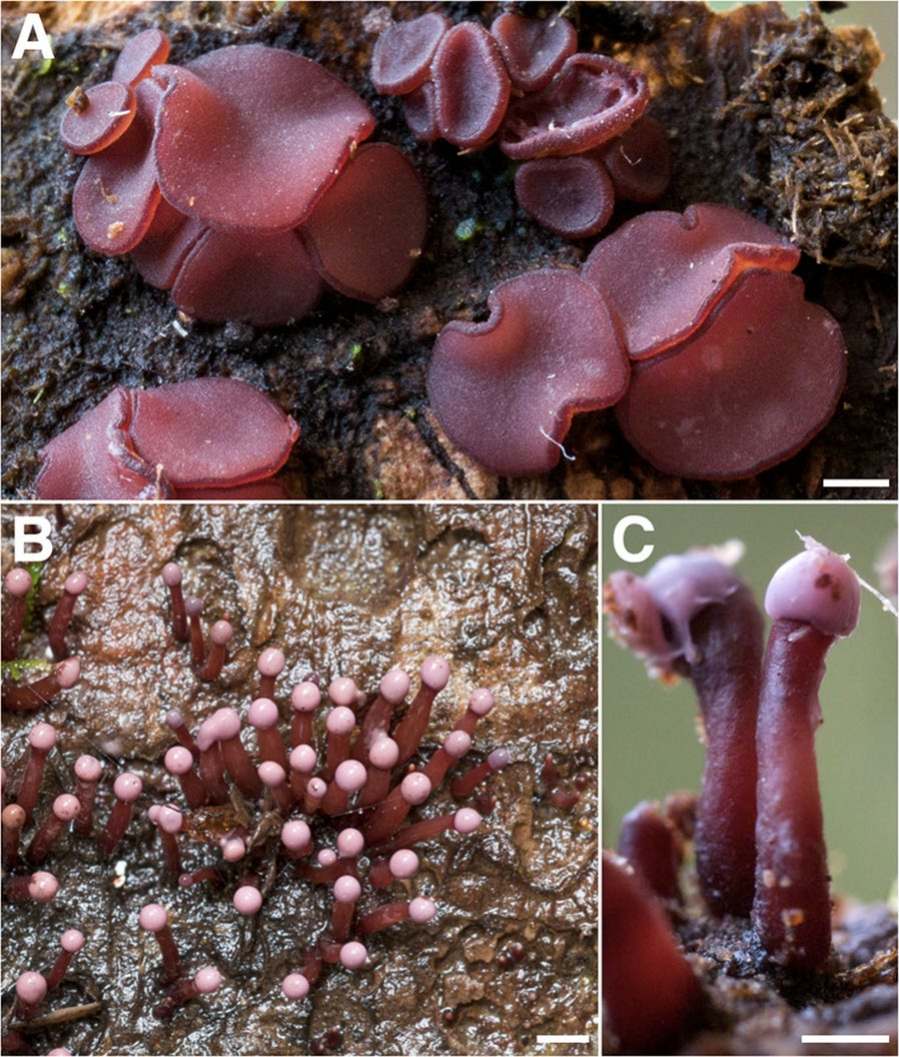
*Ascocoryne.* **A** apothecia of indet. *Ascocoryne sp.* [RLC1311]; **B**, **C** stilbelloid synnemata of *Ascocoryne cf. trichophora* [RLC1205]. Scale: A,C = 100 μm; B = 200 μm

**Fig. 11:**
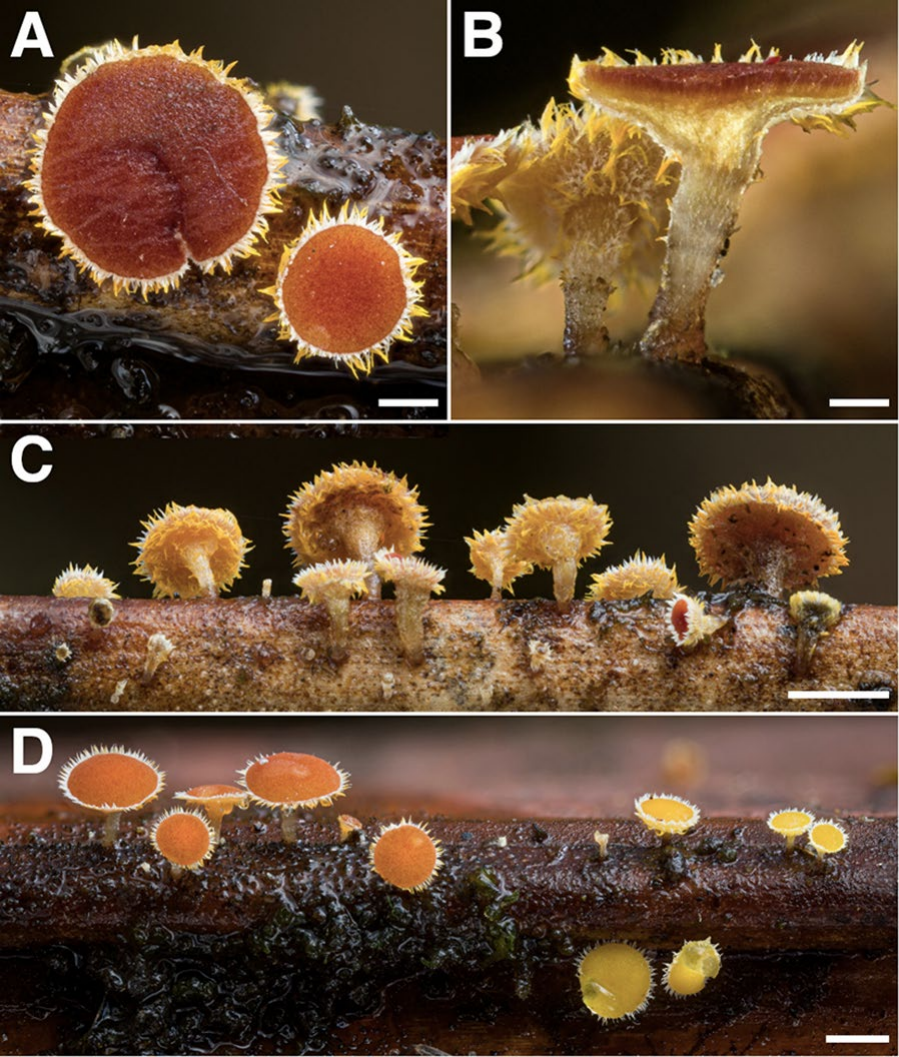
*cf. Trichopeziza*. **A** detail of crimson-colored hymenium and two-toned hairs **B** section of single fruiting body **C** detail of stipe and receptacle surfaces showing hair development along entire length **D** orange and lemon-yellow apothecia within the same collection [RLC1672] thought to represent earlier developmental stages (note lack of yellow hairs in immature fruiting bodies). Scale: A,B = 200 μm; C,D = 500 μm

*Ascocoryne cf. trichophora* (Fig. 10)—We observed and collected a purple, stilbelloid anamorph [RLC1069, 1205, 1703] which further study revealed to be a close macro- and micromorphological match to *Heydenia trichophora* A. L. Smith, described from the Dominican Republic (Smith 1901). This taxon was later recombined in *Coryne* (Seifert 1989), upon the discovery of its adjoining teleomorph,, and finally transferred to Ascocoryne (Johnston et al. 2014). ITS sequences from our Los Cedros anamorph indeed place it in the genus *Ascocoryne*, but basal to the genus’ two major clades (A. sarcoides s.l. and A. cylichnium s.l.) (Baral, unpub.), with no BLAST match exceeding 83% identity with any reference sequence in GenBank or UNITE. Unfortunately, no sequences are available from authentic material of A. trichophora. Our Ecuadorian anamorph has not been observed occurring in proximity to any teleomorph, and of those teleomorphic *Ascocoryne* collections we have made [RLC1696, 1692, 1311], none have yielded a close match to Seifert’s description of the sexual state of *Coryne* (= *Ascocoryne*)* trichophora.cf. Trichopeziza* (Fig. 11)—Our high-elevation sampling location (Fig. 1, RTG) yielded two collections [RLC1672, 1698] of a small, ornate discomycete, collected exclusively on decaying Cyclanthaceae fronds. Its combination of morphological and molecular characters enable an identification only to family level (Lachnacaeae). While possessing attributes of certain species in the genus *Trichopeziza*—such as the presence of ornamented, multiseptate, pigmented hairs which turn violet in the presence of KOH—our ITS sequence is sufficiently distant from any reference collection in that genus to merit withholding the application of this name. The genus *Belonidium* is another possibility, but this is an “old” genus in the sense of modern discomycetology, and as such is in dire need of re-circumscription, having for many decades been a “dumping ground” for superficially similar lachnoid fungi..Members of other similar, more well-defined genera in the family (e.g., *Lachnum*, *Dasyscyphella*, *Capitotricha*, *Erioscyphella*, *Lasiobelonium*, etc.) have so far failed to present a collection of characters to which the Los Cedros taxon conforms without the presence of one or more disqualifying exceptions. ITS sequences of our collections have yielded no BLAST matches within 83% identity of any publicly available sequence.

**Fig. 12:**
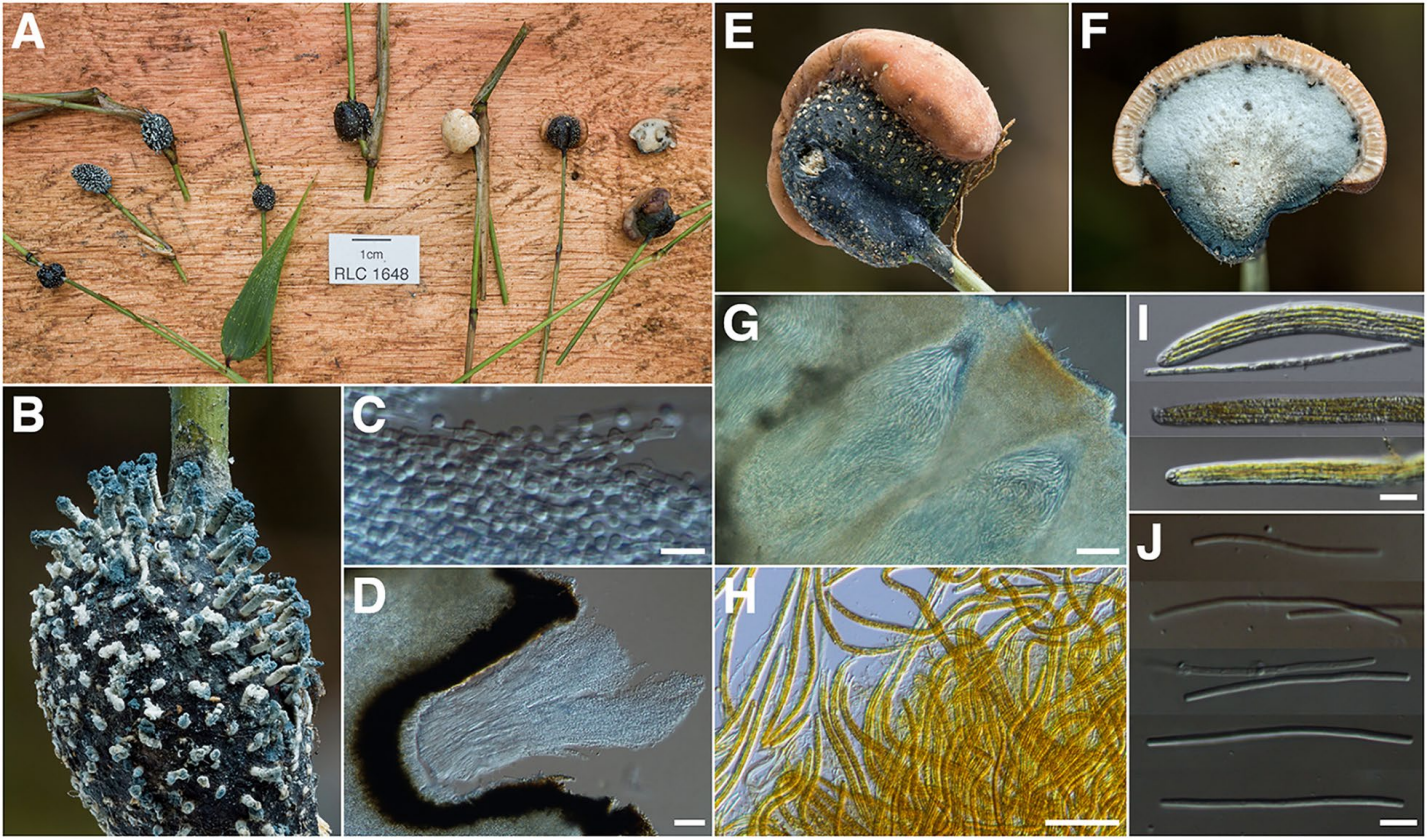
*Mycomalus/Munkia martyris.* **A** ex situ arrangement of RLC1648 containing anamorphic and pleomorphic stromata **B** close-up of sporodochial extrusions, pigmented at the leading edge **C** pleurogenous conidiophores bearing globose conidia **D** sporodochial cavity with extruding bundle of pleurogenous conidiophores **E** close-up of pleomorphic stroma showing superior *Mycomalus* teleomorphic layer (pinkish-apricot) encircling inferior sporodochial *Munkia martyris* anamorph (white dots against black) **F** cross-section of pleomorphic stroma revealing palisade of immersed perithecia (above) and some some scattered sporodochial cavities (below) **G** longitudinal section of perithecia and contents **H** asci and unejected ascospores in Lugol’s iodine solution (2.2%) **I** ascus apices **J** disarticulated part-spores. Scale: D,G,H = 10 μm; C,I,J = 50 μm

*Mycomalus* & *Munkia* (Fig. 12)—Among the many fungi described from Brazil by German mycologist Alfred Möller in his seminal work *Phycomyceten und Ascomyceten: Untersuchungen aus Brasilien* (Möller, 1901) is the monotypic *Mycomalus bambusinus*. Despite much collecting effort in the same ecoregion (Mata Atlântica) (Fidalgo 1968; Baltazar and Gibertoni 2009; Gumboski and Eliasaro 2011; Costa et al. 2014; Maia et al. 2015), this large, conspicuous fungus remains elusive.

Collections determined as *Mycomalus sp.*/*My. bambusinus* in Neotropical fungaria have been consistently found to correspond to different taxa (Newman, unpub). While recent, unconfirmed reports of the “true” *My. bambusinus* appearing in Santa Catarina province, Brazil, are awaiting authentication (Trierveiler-Pereira, pers. comm.), the holotype would appear to be the only authentic vouchered collection of the genus in the 125 years since its initial description.

This did not prevent speculation that *Mycomalus* may in fact be the sexual state of *Munkia martyris* (Spegazzini; von Höhnel 1911; Petrak 1947; Bischoff et al. 2004), a sporodochial anamorph also occurring on bamboo. Von Höhnel (von Höhnel 1911) noted the similarity between the unique conidiogenesis observed in the sporodochial cavities of *Mu. martyris* and the conidiogenesis Möller documented from germinated ascospores of *My. bambusinus*. However, the lack of pleomorphic or dimorphic collections of either genus, as well as the lack of molecular data from the *My. bambusinus* holotype, have thus far stymied efforts to determine their potential connection.

Material collected at Los Cedros finally confirms this long-standing anamorph-teleomorph hypothesis. Two collections possessing the combined attributes of *Munkia martyris* and *Mycomalus* were made during the 2018 field season, in the immediate vicinity of our expedition base camp [RLC1631, 1648]. Both collections include pleomorphic stromata, with a *Munkia martyris* anamorph and *Mycomalus* teleomorph. In addition to bringing resolution to this 122-year-old taxonomic debate, our collections also provide the first molecular data for either genus in the form of two ITS sequences (one from each collection), which are no greater than ~ 80% identical to any sequences currently in GenBank or UNITE.

Crucially, while the teleomorph we observed at Los Cedros is consistent with Möller’s generic circumscription of *Mycomalus,* it is not consistent with *My. bambusinus*, from which it differs substantially in stromatal size and color, as well as habitat and distribution (Mata Atlântica vs. Chocó). The anamorph, while being a compelling match to Spegazzini’s description of *Munkia martyris*, was originally described from low-elevation (~ 150 m.a.s.l.) Paraguayan Chaco grassland/savannah. Type studies are therefore still needed to accurately determine the identities of species involved.

Adding further intrigue is the still-unresolved question of the *Munkia/Mycomalus* nutritional mode. The amply-documented Möller genus, *Ascopolyporus*—uncannily similar to *Mycomalus* in both habit and habitat—has been shown to exhibit a unique form of dual-trophism. The fungus parasitizes insects (Coccoidea/Aleyrodoidea), which feed on living plants (mostly bamboo), consuming the insect entirely save for the stylet. The disembodied stylet is then utilized like a siphon through which the fungus extracts phloem (Bischoff et al. 2005). Transitioning hosts and nutritional modes in this way enables *Ascopolyporus* and its allies (*e.g.*, *Hypocrella, Moelleriella*, *Samuelsia, Dussiella* (= *Echinodothis*), *Neohyperdermium*) to exceed the mass of their initial insect hosts by dozens to hundreds of times. *Mycomalus* has been suspected but never demonstrated to exhibit this dual-trophic behavior (Koroch et al. 2004). Our Los Cedros material may provide a definitive and long-awaited answer to this *Mycomalus/Munkia* question as well.

**Fig. 13:**
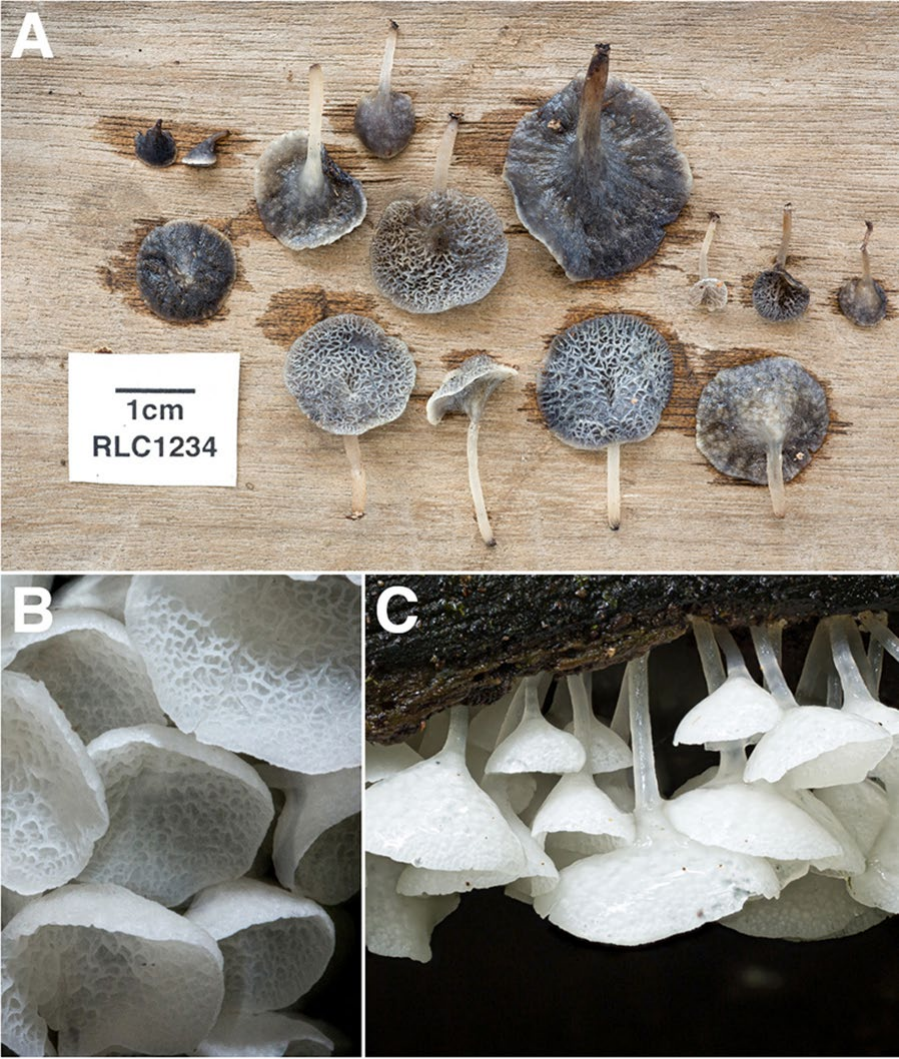
*Rhodoarrhenia.* **A** gray-blue color morph ex situ [RLC1234] **B** close-up of hymenium of white color morph [RLC1618] **C** white color morph in situ [RLC1618]

*Rhodoarrhenia* (Fig. 13)—The genus *Rhodoarrhenia* was erected by Singer in 1964 to accommodate a particular group of (sub)tropical, wood-inhabiting, reduced agarics, some of which had been previously placed by Lloyd in the genus *Rimbachia* (Singer 1963). They are characterized by a gregarious to cespitose habit, dorsally-stipitate/pendant attachment, and an anastomosing to merulioid hymenium. *Rhodoarrhenia* closely resembles descriptions and illustrations of the Pegler genus, *Skepperiella*, believed to be restricted in distribution to tropical Africa, and from which *Rhodoarrhenia* is said to differ principally in its absence of pileal and hymenial cystidia (Pegler 1973) and presence of chiastobasidia (Singer 1963). Examples of *Rhodoarrhenia* observed by us, both at Los Cedros and in cloud forests elsewhere in the Neotropics, have ranged from white to gray-blue to dingy yellow in appearance. Two such color morphs have been found to occur at Los Cedros (gray-blue and white), whose sequences share 98% ITS identity [RLC137, 813]. When joined with a multi-locus dataset including Trinidadian-Tobagoan, Guyanan, and Belizean collections, these sequences corresponded to two out four phylogenetically distinct taxa, which interestingly don’t appear to group by color (Aime, unpub). Despite being a signature fungal feature of Neotropical mountain forests, adequate circumscription of this genus is lacking, and it may be polyphyletic. Singer designated as its type a fungus with a “purplish red” spore print (*R. pezizoidea*), while all other members possess white to pale-pigmented spores (Singer 1963). On the basis of the material sequenced thus far, much of *Rhodoarrhenia* belongs squarely in the Cyphellaceae, but type studies are needed to determine what affinities *R. pezizoidea* has with these taxa. Our Los Cedros collections represent the first sequences of the genus to be uploaded to GenBank.

**Fig. 14:**
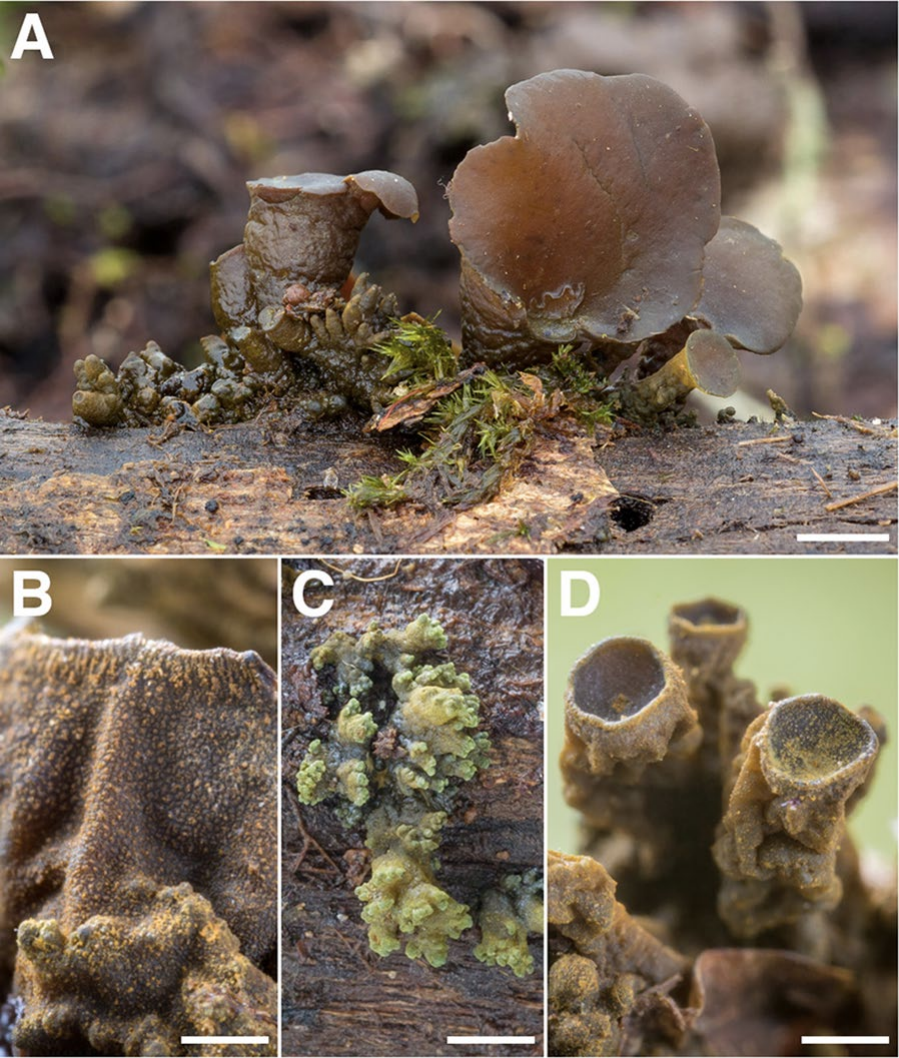
*Ionomidotis aff. fulvotingens.* **A** general habit of immature, developing and mature apothecia in situ [RLC1478] **B** close-up of orange granules **C** close-up of turquoise-tipped primordial apothecia **D** development of well-defined, fingerlike apothecia. Scale: A,C = 5 mm; B,D = 1 mm

*Ionomidotis aff. fulvotingens* (Fig. 14)—An unusual discomycete was found occurring on downed, decorticate logs in the mud and manure of the mule pasture: one of the only human-disturbed habitats within the boundary of Los Cedros. Microscopic and molecular analysis have revealed this character-rich fungus to be an undescribed and highly unique member of the *Ionomidotis fulvotingens* group, which contains several undescribed taxa (Baral, unpub.). Its ITS sequence is no greater than 89% identicalto any publicly available sequence. A review of the field photography and microcharacters of a collection from the Læssøe and Peterson expeditions of the early 2000s (TL-11793), collected less than 40 km from the Los Cedros mule pasture, suggests this collection is conspecific with our Los Cedros material. This was the second of two particularly notable collections to come from this unexpectedly prosperous habitat, the first being *Thamnomyces chocoensis* [RLC1425], each collected within days of one another in 2014, and neither observed again since.

**Fig. 15:**
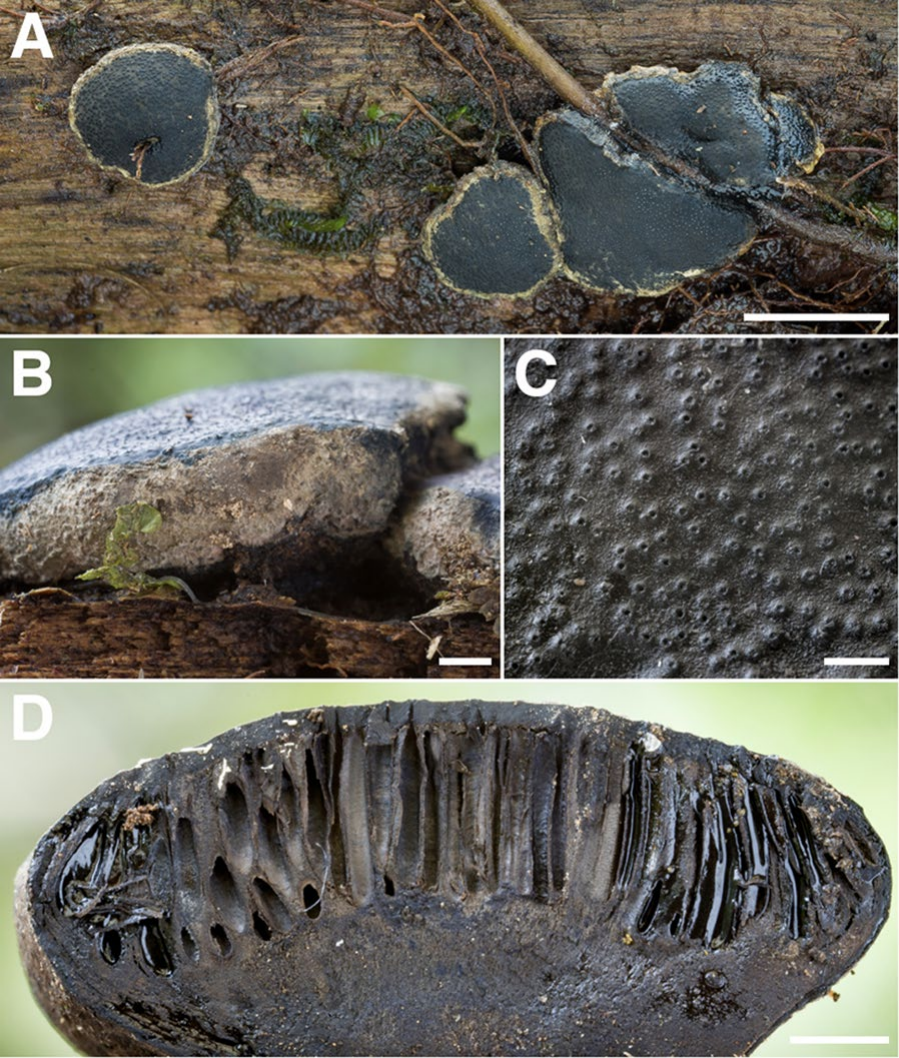
*Camarops ustulinoides.* **A** pleomorphic stromata in situ [RLC1499] **B** view of anamorphic tissue at stromatal margin **C** close-up of ostiolar mounds on stromatal surface **D** section of stroma to show elongated perithecial contents and supporting stromatal context tissue. Scale: A = 1 cm; B,D = 2 mm; C = 1 mm

*Camarops ustulinoides* (Fig. 15)—AOur 2014 expedition saw the collection of an unusual pyrenomycete, identified by Dr. Jack Rogers and Dr. Yu-Ming Ju as the rarely-reported *Camarops ustulinoides*. Despite having a somewhat xylariaceous appearance, *C. ustulinoides* does not reside in the Xylariales, but rather in the only distantly-related Boliniales (Huhndorf and Miller 2008; Untereiner et al. 2013). An ITS sequence obtained from our material [RLC1499] was found to differ from that of the only other *C. ustulinoides* ITS sequence in GenBank by almost 10%. Given the authority of the identification of our Los Cedros collection, and the somewhat opaque pedigree of this nominally conspecific reference sequence (a Puerto Rican strain purchased from a private Spanish culture library in the early 2000s), we are inclined to believe that the existing sequence is erroneously annotated.

Incidentally, the accessions with which our ITS sequence shares the highest degree of identity are three endophyte sequences taken from *Jacaranda copaia* seeds in Panama, followed closely by three endolichenic sequences taken from a collection of *Lecanora oreinoides* in Highlands, North Carolina. This would appear to make our Los Cedros collection the stromatal “face” of one or more hitherto “faceless” endophytic/endolichenic lifestyles.

**Fig. 16:**
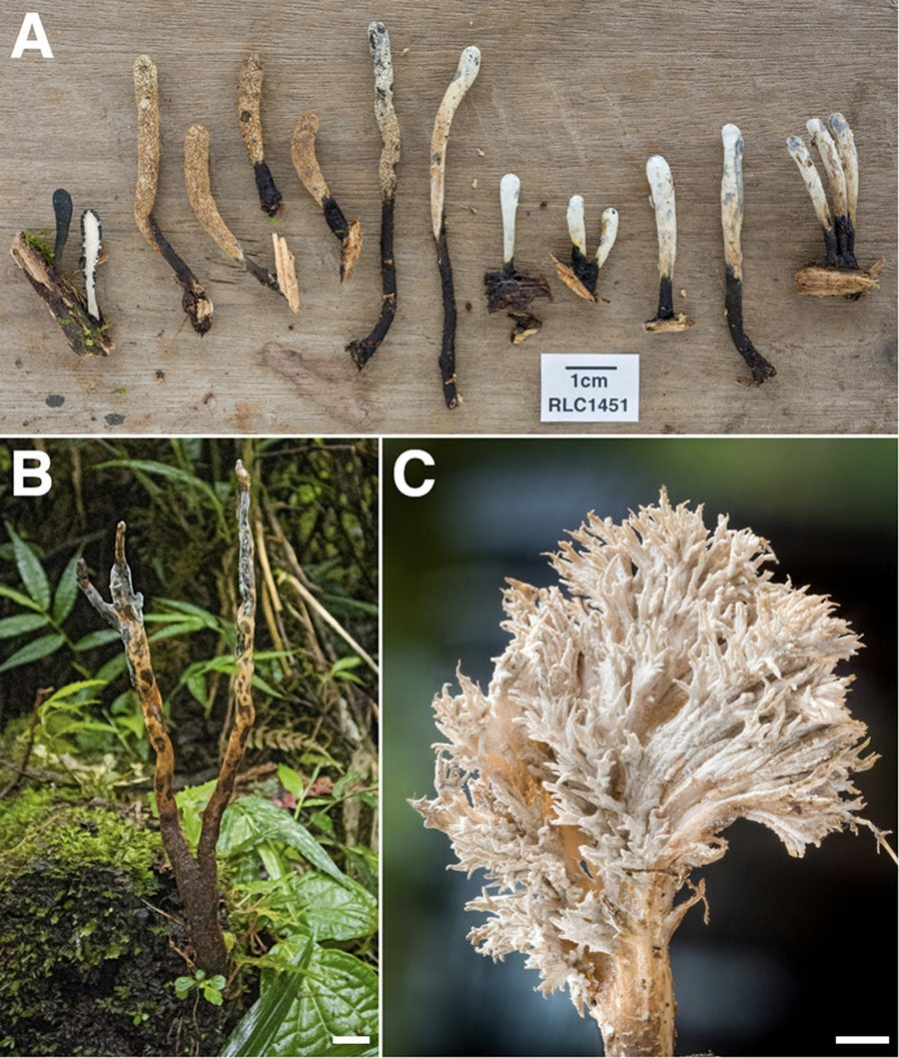
*Xylaria* spp. **A** *Xylaria sp. nov.* 02 ex situ [RLC1451] **B** *Xylaria sp. nov.* 01 in situ [RLC1829] **C** close-up of *Xylaria flabelliformis s.l.* [RLC1291]. Scale: A B C μm

*Xylaria* and Viaphytism (Fig. 16)—Collections of the genus *Xylaria* from Los Cedros have been used to elucidate a novel ecological theory, known as the Foraging Ascomycete Hypothesis, or simply viaphytism (Thomas et al. 2016, 2020; Nelson et al. 2020). Briefly, viaphytism refers to the utilization of a leaf-endophytic life stage by typically saprobic fungi as a means of dispersal; the fungi travel by way of (“via-”) the plants’ leaves (“-phyte”) to bridge spatial and temporal gaps in preferred substrate. This allows for persistence in the environment, even when substrates or environmental conditions that allow fruiting are absent.

Samples targeting the genus within the Permanent Forest Plot were collected in 2012 in combination with extensive cultivation of endophytic fungi from the leaves of trees within the plot; ITS sequences were used to associate collected stromata with endophyte occurrence, and permutational nearest-neighbor analysis was used to examine spatial co-occurrence of the two life stages (Thomas et al. 2016). From that experiment, there was only a single taxon found occurring as an endophyte that was not also found fruiting on the forest floor within the plot, *Xylaria flabelliformis* s.l., a species with a distinctive anamorph; it has since been collected several times elsewhere at Los Cedros [RLC220, 643, 1301, 1407, 1291] (Fig. 16c).

That study, which provided the first concrete evidence of the viaphytic lifestyle, recorded 36 species of *Xylaria* from Los Cedros, of which 19 could be confidently assigned to named species; here, we emend that a total of at least 55 putative species of *Xylaria*, of which 25 can be confidently assigned to a named species, as well as three undescribed taxa. Among these is one particularly charismatic *Xylaria* sp. nov. [RLC1126, 1129, 1429, 1711, 1828, 1829] (Fig. 16b), which possesses some of the longest stromata ever recorded in the genus (≥ 25 cm). A second putative *Xylaria* sp. nov. [RLC1334, 1451] (Fig. 16a; Additional file 2, “*Xylaria* sp. nov. 02”) represents one of the few taxa in the world known to occur on bamboo, the substrate from which both collections of that species were made.

### Conservation in action

**Fig. 17:**
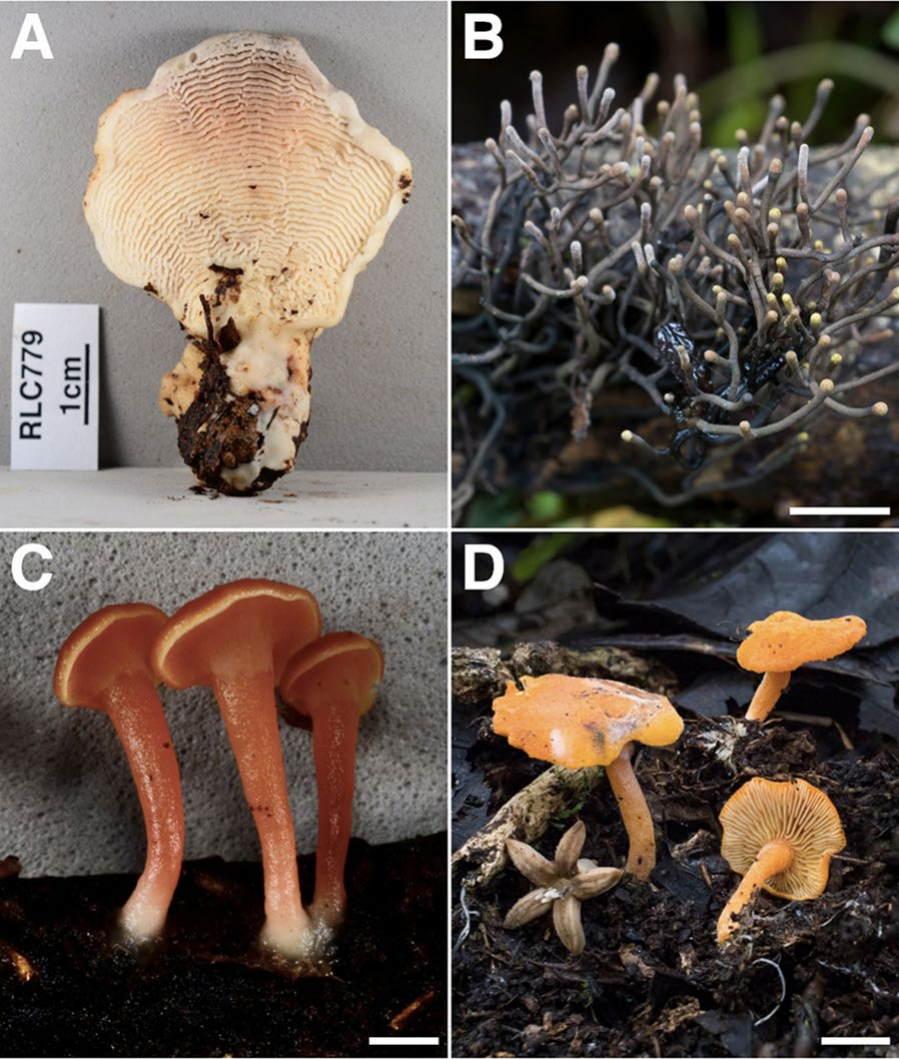
RLC Taxa submitted for IUCN Global Fungal Red List assessment. **A** *Lamelloporus americanus* [RLC779] **B** *Thamnomyces chocoensis* [RLC1425] **C** *Hygrocybe aphylla* [RLC740] **D** *“Lactocollybia” aurantiaca* [RLC1839]. Scale: B,D = 1 cm; C = 2 mm

Among our Los Cedros collections are four species nominated to the IUCN Global Fungal Red List Initiative (Dahlberg and Mueller 2011): *Lamelloporus americanus*, *Thamnomyces chocoensis*, *Hygrocybe aphylla*, and *“Lactocollybia” aurantiaca* (Fig. 17); all are awaiting final assessment. Their distributions range from the broadly Neotropical (*H. aphylla* & “*L.” aurantiaca*) to apparent endemics (*L. americanus* & *T. chocoensis*), with *T. chocoensis* known only from the holotype collection and our Los Cedros material, collected less than 80 km apart from one another. We suspect many of the undescribed taxa encountered at Los Cedros to be unique to the Chocò bioregion, an area known for high levels of endemicity (Gentry 1992; Myers et al. 2000; Quijano-Abril et al. 2006; Ruiz-Guerra et al. 2007; Frahm 2012). As such, we intend to submit any newly described taxa for IUCN assessment as they are published.

Such designations are likely to be of significant value to future conservation initiatives, at Los Cedros or any similarly threatened forest where the same species are shown to occur. This is directly evidenced by the citing of these IUCN-nominated taxa by the constitutional court in its written decision (Jiménez 2021), which references Los Cedros’ funga in three of its 105 enumerated sections. This is the first time fungal diversity data has impacted an Ecuadorian constitutional court judgment, and the second instance on the South American continent of fungal diversity and conservation reaching federal levels of deliberation. The first was the passage of legislation in the Chilean Parliament granting equal recognition and protection to funga under the law as was guaranteed to flora and fauna (Biblioteca del Congreso Nacional de Chile 2022); a landmark achievement of the now multinational NGO, Fundación Fungi, and its foundress, Giuliana Furci.

Pioneers at the still largely-uncharted frontier of fungal conservation have highlighted the importance of biodiversity and phenological data as foundational first steps toward obtaining a comprehensive picture of the funga of a given locality, such that it may form a meaningful part of the conservation conversation (Dahlberg et al. 2010; Gonçalves et al. 2021; Mueller et al. 2022). Less demonstrated or discussed, however, is the political potency these baseline metrics possess in the defending of habitats from damage or destruction posed by major agribusiness, urban development, extractive industry, and other existential threats.

We present our ongoing work at Los Cedros as a case study in biodiversity research as conservational praxis, in the hopes that fellow biologists may recognize the power of their own data to promote change in environmental decision-making, even at the highest levels of government.

## Supplementary Information

**Additional file 1:** Taxonomic list, structured hierarchically, of all vouchered fungi and fungus-like organisms from Los Cedros.

**Additional file 2:** Collection data for all vouchered specimens across the entire study, structured by individual collection.

**Additional file 3:** Data from vouchered collections used in the 2010 ecological experiment, and the taxonomic list derived therefrom.

## Data Availability

All data and scripts used in the preparation of this manuscript are available in Additional file 1: Appendix S1, Additional file 2: Appendix S2, Additional file 3: Appendix S3, or the FigShare repository Vandegrift R, Newman DS, Dentinger B, et al. (2023) Data from: Richer than Gold: the fungal biodiversity of Reserva Los Cedros, a threatened Andean cloud forest. Figshare. 10. 6084/m9.figshare.22043828.
