# Supplementary material for: Richer than Gold: the fungal biodiversity of Reserva Los Cedros, a threatened Andean cloud forest
Source: Bot Stud. 2023 Jul 6;64:17. doi: 10.1186/s40529-023-00390-z (PMC10326184; doi:10.1186/s40529-023-00390-z)
Supplement: Supplementary file 1 — Additional file 1: Taxonomic list, structured hierarchically, of all vouchered fungi and fungus-like organisms from Los Cedros. [file 40529_2023_390_MOESM1_ESM.pdf]

Appendix 1: Taxonomic list of fungi and fungus-like organisms (Mycetozoa) recorded within the Reserva Los Cedros, including a few incidental lichen collections.. Listed are PHYLUM, then indented class, order, and family, followed by taxon identity at the genus and species level, along with authorities for taxa identified at the species level. Where multiple OTUs (at 97% similarity; see *Methods*) are present within the same taxonomic determination, the number of OTUs are noted in square brackets after the taxon name. We have been conservative with the application of names for taxa in groups for which we are not specialists; see *Methods* for an explanation of our criteria for application of open nomenclature qualifiers. The number of collections with a final determination for any given level are listed to the right (note: this is *not* the total number of collections within a given rank, but the number of collections with that rank as its most precise determination); not included are the 45 collections determined only as Fungi. Taxa identified at higher than genus-level using the qualifier 'sensu lato' are counted with the next rank above (*e.g.*, a collection identified as "Agaricales s.l." is counted with "Agaricomycetes"). For information organized by collection, including collection and accession numbers, please see Appendix 2.

AMOEBOZOA (Mycetozoa)

Myxogastria	8
Liceales	
Tubiferaceae	
Tubifera microsperma (Berk. & M.A. Curtis) G.W. Martin	1
Physarales	
Didymiaceae	
Diachea cf. *leucopodia* (Bull.) Rostaf.	2
Diachea sp.	2
Diderma cf. *effusum* (Schwein.) Morgan	1
cf. *Didymium* sp.	1
Physaraceae	
cf. *Physarum* sp.	1
Stemonitales	
Stemonitidaceae	
cf. *Stemonitis* sp.	1
Trichiales	
Arcyriaceae	
Arcyria cf. *cinerea* (Bull.) Pers.	1
Arcyria sp.	1
Protosteliomycetes	
Protosteliales	
Ceratiomyxaceae	
Ceratiomyxa fruticulosa (O.F. Müll.) T. Macbr.	1
Ceratiomyxa morchella A.L. Welden	1
Ceratiomyxa sphaerosperma Boedijn	1

ASCOMYCOTA **24**

- Dothidiomycetes
  - Pleosporales
    - Roussoellaceae 1

- Eurotiomycetes
  - Eurotiales
    - Trichocomaceae
      - *Talaromyces palmae* (Samson, Stolk & Frisvad) Samson, Yilmaz, Frisvad & Seifert 1
      - cf. *Talaromyces* sp. 1
  - Pyrenulales
    - Pyrenulaceae
      - *Pyrenula* sp. 1

- Laboulbeniomycetes
  - Laboulbeniales
    - Laboulbeniaceae
      - *Laboulbenia* sp. 1

- Lecanoromycetes
  - Baeomycetales
    - Baeomycetaceae
      - *Phyllobaeis* sp. 1
  - Peltigerales
    - Collemataceae
      - *Leptogium* sp. 2
    - Lobariaceae
      - *Sticta weigelii* (Ach.) Vain. 1
  - Teloschistales
    - Caliciaceae 1

- Leotiomycetes 1
  - Helotiales 4
    - Cordieritidaceae 3
      - *Cordierites coralloides* Berk. & M.A. Curtis 1
      - *Encoelia heteromera* (Mont.) Nannf. 2
      - *Ionomidotis fulvotingens* group (sp. nov.) (Berk. & M.A. Curtis) E.K. Cash 1
    - Dermateaceae
      - cf. *Niptera* sp. 1
    - Gelatinodiscaceae
      - *Ascocoryne cylichnium* (Tul.) Korf 1
      - *Ascocoryne sarcoides* complex (Jacq.) J.W. Groves & D.E. Wilson 1
      - *Ascocoryne* cf. *trichophora* (A.L. Sm.) Seifert 1
      - *Ascocoryne* sp. 1
      - *Neobulgaria* sp. [3x OTUs] 4
      - cf. *Neobulgaria* sp. 1
    - Helotiaceae
      - *Dicephalospora albolutea* H.D. Zheng & W.Y. Zhuang 1

Dicephalospora rufocornea (Berk. & Broome) Spooner	1
Dicephalospora sp.	1
Lachnaceae [4x OTUs]	7
cf. *Trichopeziza* sp.	2
Erioscyphella brasiliensis (Mont.) Baral, Šandová & B. Perić	2
Erioscyphella cf. *brasiliensis* (Mont.) Baral, Šandová & B. Perić	1
Mollisiaceae	1
Pezizellaceae	
Calycina sp.	1
Rutstroemiaceae	
Lanzia sp.	1
Sclerotiniaceae	2
Rhytismatales	1
Pezizomycetes	
Pezizales	
Pyronemataceae	
Scutellinia sp.	2
Sphaerosporium cf. *lignatile* Schwein.	1
Sarcoscyphaceae	
Cookeina tricholoma (Mont.) Kuntze	4
Phillipsia domingensis (Berk.) Berk. ex Denison	1
Wynneaceae	
Wynnea americana Thaxt.	1
Wynnea cf. *gigantea* Berk. & M.A. Curtis	2
Sordariomycetes	11
Boliniales	
Boliniaceae	
Camarops ustulinoides (Henn.) Nannf.	1
Diaporthales	
Cryphonectriaceae	
Aurapex penicilliata Gryzenhout & M.J. Wingf.	2
Hypocreales sp.	4
Bionectriaceae	2
Clavicipitaceae [2x OTUs]	14
Keithomyces sp.	1
Moelleriella turbinata (Petch) P. Chaverri & K.T. Hodge	1
cf. *Moelleriella* sp.	1
Mycomalus sp.	2
Nigelia martialis (Speg.) Luangsa-ard & Thanakitp.	3
aff. *Polyocephalomyces* sp.	1
Torrubiella sp.	2
cf. *Torrubiella* sp.	1
Cordycipitaceae	14
Akanthomyces tuberculatus complex (Lebert) Spatafora, Kepler & B. Shrestha	3
Ascopolyporus polychrous Möller	6
Ascopolyporus villosus Möller	1

Beauveria cf. *loeiensis* Luangsa-ard, Ridkaew & Tasanathai	1
Beauveria sp.	2
cf. *Conoideocrella* sp.	1
Cordyceps cf. *caloceroides* Berk. & M.A. Curtis	1
Cordyceps nidus complex T. Sanjuan, Chir.-Salom. & S. Restrepo	8
Cordyceps pruinosa group Petch	1
Cordyceps spegazzinii M.S. Torres, J.F. White & J.F. Bisch.	2
Cordyceps cf. *spegazzinii* M.S. Torres, J.F. White & J.F. Bisch.	2
Cordyceps tenuipes (Peck) Kepler, B. Shrestha & Spatafora	5
Cordyceps sp.	7
Cordyceps sensu lato	4
Gibellula sp.	1
Isaria sp.	4
cf. *Metacordyceps* sp.	1
Neohyperdermium piperis (J.F. Bisch. & J.F. White) Thanakitp. & Luangsa-ard	1
cf. *Neohyperdermium* sp.	1
Hypocreaceae	
Hypomyces tremellicola (Ellis & Everh.) Rogerson	2
Trichoderma sp.	2
cf. *Trichoderma* sp. [2x OTUs]	2
Nectriaceae	
Cosmospora sp.	1
Nectria cinnabarina complex (Tode) Fr.	1
cf. *Neocosmospora* sp.	1
Pseudocosmospora sp.	1
Ophiocordycipitaceae	1
Ophiocordyceps australis s.l. (Speg.) G.H. Sung, J.M. Sung, Hywel-Jones & Spatafora	1
Ophiocordyceps curculionum (Tul. & C. Tul.) G.H. Sung, J.M. Sung, Hywel-Jones & Spatafora	1
Ophiocordyceps humbertii (C.P. Robin) G.H. Sung, J.M. Sung, Hywel-Jones & Spatafora	1
Ophiocordyceps melolonthae (Tul. & C. Tul.) G.H. Sung, J.M. Sung, Hywel-Jones & Spatafora	1
Ophiocordyceps cf. *nutans* (Pat.) G.H. Sung, J.M. Sung, Hywel-Jones & Spatafora	4
Ophiocordyceps cf. *sphecocephala* (Klotzsch ex Berk.) G.H. Sung, J.M. Sung, Hywel-Jones & Spatafora	1
Ophiocordyceps unilaterlis group (Tul.) Petch	1
Ophiocordyceps sp.	1
Purpureocillium atypicola (Yasuda) Spatafora, Hywel-Jones & Luangsa-ard	1
Purpureocillium sp.	2
Ophiostomatales	1
Ophiostomataceae	
Ophiostoma epigloeum (Guerrero) de Hoog	1
Ophiostoma sp.	1
Sordariales	
Nitschkiaceae	
cf. *Bertia* sp.	1
Sphaeriales	
Sphaeriaceae	

Stromatographium stromaticum (Berk.) Höhn.	3
Xylariales	5
Graphostromataceae	
Biscogniauxia cf. *petrensis* Z.F. Zhang, F. Liu & L. Cai	1
Biscogniauxia sp.	7
Camillea oligoporus Læssøe, J.D. Rogers & Whalley	1
Hypoxylaceae	1
Annulohypoxylon aff. *leptascum* (Speg.) Y.M. Ju, J.D. Rogers & H.M. Hsieh	1
Annulohypoxylon stygium (Lév.) Y.M. Ju, J.D. Rogers & H.M. Hsieh	1
Annulohypoxylon subeffusum (Speg.) Hladki & A.I. Romero	1
Annulohypoxylon cf. *substygium* Sir & Kuhnert	1
Annulohypoxylon sp. [7x OTUs]	11
Daldinia eschscholtzii (Ehrenb.) Rehm	1
Hypoxylon trugodes Berk. & Broome	2
Hypoxylon sp.	5
Phylacia poculiformis (Kunze) Mont.	1
Pyrenopolyporus symphyon (Möller) M. Stadler, Kuhnert & L. Wendt	2
cf. *Pyrenopolyporus* sp.	1
Thamnomyces chocoënsis Læssøe	1
Thamnomyces cf. *rostratus* Mont.	2
Xylariaceae	13
Kretzschmaria clavus (Fr.) Sacc.	6
Kretzschmaria aff. *clavus* (Fr.) Sacc.	2
Kretzschmaria cf. *clavus* (Fr.) Sacc.	1
Kretzschmaria lucidula (Mont.) Dennis	3
Kretzschmaria aff. *lucidula* (Mont.) Dennis	1
Kretzschmaria milleri J.D. Rogers & Y.M. Ju	2
Kretzschmaria cf. *milleri* J.D. Rogers & Y.M. Ju	1
Kretzschmaria cf. *pavimentosa* (Ces.) P.M.D. Martin	3
Kretzschmaria zonata (Lév.) P.M.D. Martin	1
Kretzschmaria cf. *zonata* (Lév.) P.M.D. Martin	1
Kretzschmaria sp. [2x OTUs]	7
Kretzschmaria sp. nov.	1
cf. *Kretzschmariella culmorum* (Cooke) Y.M. Ju & J.D. Rogers	1
Nemania bipapillata (Berk. & M.A. Curtis) Pouzar	4
Nemania cf. *bipapillata* (Berk. & M.A. Curtis) Pouzar	1
Nemania diffusa (Sowerby) Gray	3
Nemania cf. *diffusa* (Sowerby) Gray	1
Nemania sp. [2x OTUs]	5
Rosellinia perusensis Henn.	1
Rosellinia sp.	4
cf. *Thuemenella* sp.	1
Xylaria adscendens (Fr.) Fr.	6
Xylaria aff. *adscendens* (Fr.) Fr.	1
Xylaria anisopleura (Mont.) Fr.	8
Xylaria anisopleura group (Mont.) Fr.	1
Xylaria apiculata Cooke	21
Xylaria apiculata group Cooke	1

Xylaria atrosphaerica (Cooke & Massee) Callan & J.D. Rogers	5
Xylaria cf. *atrosphaerica* (Cooke & Massee) Callan & J.D. Rogers	2
Xylaria berteroi (Mont.) Cooke ex J.D. Rogers & Y.M. Ju	2
Xylaria clusiae K.F. Rodrigues, J.D. Rogers & Samuels	1
Xylaria comosa complex (Mont.) Mont.	2
Xylaria aff. *comosa* (Mont.) Mont. (sensu Læssøe 1999)	12
Xylaria corniculata Sacc.	3
Xylaria cristata Speg.	1
Xylaria cf. *culicicephala* A.I. Romero & Hladki	1
Xylaria cuneata Lloyd	6
Xylaria curta Fr. [2x OTUs]	4
Xylaria aff. *curta* Fr.	4
Xylaria enterogena Mont.	25
Xylaria cf. *enterogena* Mont.	1
Xylaria fissilis Ces. [2x OTUs]	43
Xylaria aff. *fissilis* Ces.	4
Xylaria cf. *fissilis* Ces.	1
Xylaria flabelliformis s.l. (Schwein.) Berk. & M.A. Curtis	5
Xylaria globosa (Spreng.) Mont.	17
Xylaria cf. *gracillima* (Fr.) Fr.	1
Xylaria cf. *heliscus* (Mont.) J.D. Rogers & Y.M. Ju	1
Xylaria ianthinovelutina (Mont.) Mont.	3
Xylaria kegeliana (Lév.) Fr.	2
Xylaria cf. *kegeliana* (Lév.) Fr.	2
Xylaria melanura (Lév.) Sacc.	1
Xylaria melanura group (Lév.) Sacc.	3
Xylaria meliacearum Læssøe	4
Xylaria cf. *meliacearum* Læssøe	1
Xylaria multiplex (Kunze ex Fr.) Fr.	8
Xylaria cf. *obovata* (Berk.) Berk.	1
Xylaria schweinitzii Berk. & M.A. Curtis [2x OTUs]	41
Xylaria cf. *schweinitzii* Berk. & M.A. Curtis	1
Xylaria scruposa (Fr.) Fr. [2x OTUs]	31
Xylaria aff. *scruposa* (Fr.) Fr.	1
Xylaria subtorulosa Speg.	3
Xylaria cf. *subtorulosa* Speg.	1
Xylaria telfairii (Berk.) Sacc.	18
Xylaria cf. *telfairii* (Berk.) Sacc.	1
Xylaria tuberoides Rehm	4
Xylaria sp. [19x OTUs]	99
Xylaria sp. nov. 01	6
Xylaria sp. nov. 02	2
Xylaria sp. nov. 03	2
Xylobotryomycetes	
Xylobotryales	
Xylobotryaceae	
Xylobotryum andinum Pat.	1

Xylobotryum portentosum (Mont.) Pat. [2x OTUs]	5
Xylobotryum sp.	3
**BASIDIOMYCOTA**	**4**
Agaricomycetes	32
Agaricales	95
Agaricaceae	2
Agaricus butyreburneus Kerrigan, Guinb. & Callac	1
Agaricus lodgeae L.A. Parra, Angelini & B. Ortiz	1
Agaricus microincrustatus L.A. Parra, B. Ortiz, Lodge & T.J. Baroni	2
Agaricus aff. *parvibicolor* Linda J. Chen, R.L. Zhao & K.D. Hyde	1
Agaricus sp.	4
cf. *Cystolepiota* sp. [2x OTUs]	2
Lepiota s.l. sp. [7x OTUs]	13
Leucoagaricus cf. *gongylophorus* (Möller) Singer	1
Leucoagaricus sp. [4x OTUs]	5
cf. *Leucoagaricus* sp.	1
Leucoagaricus viridiflavus (Petch) T.K.A. Kumar & Manim.	1
Leucoagaricus cf. *viridiflavus* (Petch) T.K.A. Kumar & Manim.	1
Leucocoprinus cf. *brunneoluteus* Capelari & Gimenes	1
Leucocoprinus cf. *cepistipes* (Sowerby) Pat.	2
Leucocoprinus sp. [8x OTUs]	8
Lycoperdon sp. [3x OTUs]	9
Callistosporiaceae	
Callistosporium sp.	1
Clavariaceae	
Clavaria cf. *acuta* Sowerby	1
Ramariopsis sp. [2x OTUs]	4
Scytinopogon sp.	1
Clavulinaceae	
Clavulinopsis sp.	1
Clitocybaceae	
Singerocybe sp.	3
Cortinariaceae	
Pyrrhoglossum sp.	1
Crepidotaceae	
Crepidotus sp. [6x OTUs]	14
aff. *Crepidotus* sp. 01	1
Cyphellaceae	
Rhodoarrhenia sp.	4
Cyphellopsidaceae	
Calathella cf. *columbiana* Agerer	2
Lachnella sp.	2
Entolomataceae [2x OTUs]	4
Clitopilus sp.	1
Entoloma belouvense Noordel. & Hauskn.	1
Entoloma caeruleomarginatum Reschke, Manz & Noordel.	1

Entoloma subg. *Nolanea* sp.	4
Entoloma subg. *Claudopus* sp.	1
Entoloma sp. [6x OTUs]	8
Rhodocybe sp.	1
Hygrophoraceae	2
Chrysomphalina sp. 01	2
Cyphellostereum pusiolum (Berk. & M.A. Curtis) D.A. Reid	1
Dictyonema aff. *barbatum* Dal-Forno, Bungartz & Lücking	1
Hygrocybe aphylla Læssøe & Boertm.	1
Hygrocybe aff. *astatogala* (R. Heim) Heinem.	2
Hygrocybe hypohaemacta (sensu auct. neotrop.) (Corner) Pegler	1
Hygrocybe aff. *miniata* (Fr.) P. Kumm.	1
Hygrocybe nigrescens group (Quél.) Kühner	1
Hygrocybe occidentalis complex (Dennis) Pegler	5
Hygrocybe cf. *rosea* Murrill	2
Hygrocybe sect. *Psuedofirmae* sp.	2
Hygrocybe sp. [4x OTUs]	7
Rimbachia paradoxa Pat.	1
Hymenogastraceae	
Galerina sp. [3x OTUs]	5
cf. *Galerina* sp.	1
Gymnopilus lepidotus Hesler	1
Gymnopilus sp. [2x OTUs]	3
Phaeocollybia sp.	2
Psilocybe guilartensis Guzmán, F. Tapia & Nieves-Riv.	3
Psilocybe yungensis Singer & A.H. Sm.	2
Psilocybe zapotecorum R. Heim	4
Psilocybe sect. *Cordisporae* sp.	3
Psilocybe sp.	1
Psilocybe sp. nov.	2
Lyophyllaceae	
cf. *Ossicaulis* sp. 01	3
Marasmiaceae	1
Amyloflagellula sp.	1
Campanella sp.	2
Crinipellis sp.	2
Hymenogloea papyracea (Berk. & M.A. Curtis) Singer	2
Hymenogloea cf. *papyracea* (Berk. & M.A. Curtis) Singer	1
Marasmius aff. *cladophyllus* Berk.	1
Marasmius cladophyllus Berk.	2
Marasmius cf. *cladophyllus* Berk.	1
Marasmius congregatus Mont.	4
Marasmius griseoroseus (Mont.) Singer	2
Marasmius haematocephalus (Mont.) Fr.	1
Marasmius sect. *Globulares* sp.	1
Marasmius sect. *Marasmius* sp.	3
Marasmius sp. [14x OTUs]	19
Tetrapyrgos sp.	1

Mycenaceae	15
Favolaschia sp. [3x OTUs]	8
Filoboletus cf. *gracilis* (Klotzsch ex Berk.) Singer [2x OTUs]	13
Filoboletus sp.	1
Mycena chloroxantha Singer [2x OTUs]	11
Mycena cf. *discobasis* Métrod	1
Mycena sect. *Calodontes* sp.	1
Mycena sect. *Exornatae* sp.	2
Mycena sect. *Longisetae* sp.	2
Mycena sect. *Sacchariferae* sp.	2
Mycena sp. [27x OTUs]	66
cf. *Mycena* sp. [2x OTUs]	6
Mycena sensu lato	1
Panellus cf. *pusillus* (Pers. ex Lév.) Burds. & O.K. Mill.	5
Panellus sp.	1
cf. *Pleurotopsis* sp.	1
Resinomycena sp.	2
cf. *Resinomycena* sp. 01	6
Roridomyces sp.	1
Niaceae	
cf. *Flagelloscypha* sp.	1
Nidulariaceae	
Cyathus sect. *Eucyathus* sp.	2
Cyathus sp.	1
Omphalotaceae [6x OTUs]	7
Collybiopsis cf. *subcyathiformis* (Murrill) R.H. Petersen	1
Gymnopus aff. *macropus* Halling	1
Gymnopus omphalodes (Berk.) Halling & J.L. Mata	3
Gymnopus aff. *pseudolodgeae* J.L. Mata	1
cf. *Gymnopus* sp.	2
Gymnopus sp. [10x OTUs]	17
Marasmiellus sp. [4x OTUs]	4
Marasmiellus aff. *volvatus* Singer	2
Mycetinis sp.	1
cf. *Pusillomyces* sp.	1
Rhodocollybia amica J.L. Mata & Halling	2
Rhodocollybia sp. [3x OTUs]	4
Phyllotopsidaceae	
Tricholomopsis aurea (Beeli) Desjardin & B.A. Perry	2
Tricholomopsis sp. [6x OTUs]	11
Physalacriaceae [4x OTUs]	7
Armillaria puiggarii Speg.	1
Armillaria cf. *puiggarii* Speg.	3
Armillaria sp. [2x OTUs]	4
Cyptotrama sp.	3
cf. *Cyptotrama* sp.	1
Gloiocephala sp. [2x OTUs]	5
Hymenopellis sp.	1

- *Oudemansiella canarii* (Jungh.) Höhn. 1
- *Oudemansiella* sp. 1
- *Physalacria* sp. 3
- *Physalacria* s.l. 2
- *Pseudohiatula* sp. biocode09-464/467/468 (sensu Osmundson et al.; crypt. temp.) 1

Pleurotaceae
- *Hohenbuehelia* sp. [2x OTUs] 6
- *Pleurotus albidus* (Berk.) Pegler 1
- *Pleurotus* sp. 5
- *Resupinatus* sp. 1
- cf. *Resupinatus* sp. 1

Pluteaceae
- *Pluteus aureovenatus* complex Menolli & Capelari 3
- *Pluteus* sp. [3x OTUs] 6

Porotheleaceae [2x OTUs] 2
- *Clitocybula* sp. [2x OTUs] 3
- *Gerronema* sp. 1
- *Hydropus* cf. *cavipes* (Pat. & Gaillard) Dennis 1
- *Hydropus* cf. *nigrita* (Berk. & M.A. Curtis) Singer 2
- *Hydropus* sp. TL1151 (sensu Læssøe & Petersen) 1
- *Hydropus* sp. [2x OTUs] 3
- cf. *Hydropus* sp. 1
- cf. *Lactocollybia* sp. 1
- *Phloeomana* sp. [2x OTUs] 2
- cf. *Rectipilus* sp. 1
- cf. *Trogia* sp. 1

Psathyrellaceae 1
- *Candolleomyces* sp. [2x OTUs] 2
- *Coprinellus disseminatus* complex (Pers.) J.E. Lange 4
- *Coprinellus disseminatus* complex (America clade) (Pers.) J.E. Lange 3
- *Panaeolus* sp. 1
- *Parasola* sp. [2x OTUs] 2
- *Psathyrella* cf. *bipellis* (Quél.) A.H. Sm. 1
- *Psathyrella* cf. *maculata* (C.S. Parker) A.H. Sm. 1
- *Psathyrella* sp. [2x OTUs] 5
- *Tulosesus* sp. 1

Pterulaceae 6
- *Phaeopterula* sp. 3
- *Pterula* cf. *loretensis* Corner 1
- *Pterula* sp. 2
- *Pterulicium* cf. *caricis-pendulae* (Corner) Leal-Dutra, Dentinger & G.W. Griff. 1
- *Pterulicium* aff. *echo* (D.J. McLaughlin & E.G. McLaughlin) Leal-Dutra, Dentinger & G.W. Griff. 2
- *Pterulicium* cf. *echo* (D.J. McLaughlin & E.G. McLaughlin) Leal-Dutra, Dentinger & G.W. Griff. 1
- *Pterulicium* cf. *secundirameum* (Lév.) Leal-Dutra, Dentinger & G.W. Griff. 1
- *Pterulicium* aff. *subsimplex* (Henn.) Leal-Dutra, Dentinger & G.W. Griff. 2
- *Pterulicium* cf. *subsimplex* (Henn.) Leal-Dutra, Dentinger & G.W. Griff. 5

cf. *Pterulicium* sp.	1
Pterulicium sp. [8x OTUs]	23
Strophariaceae	
Deconica coprophila (Bull.) P. Karst.	1
Deconica sp. [2x OTUs]	2
Deconica sp. (melanotoid) [2x OTUs]	3
Hypholoma sp. [3x OTUs]	3
Kuehneromyces sp.	1
Pholiota nubicola (Singer) Matheny & P.-A. Moreau	3
Pholiota sp.	2
Stropharia sp.	1
Tricholomataceae	
cf. *Pseudobaeospora* sp.	1
Tubariaceae	
Tubaria sp.	1
Typhulaceae	
Macrotyphula sp. [2x OTUs]	4
Typhula sp.	2
cf. *Typhula* sp.	1
Typhula s.l.	1
unassigned sect. Marasmiineae [3x OTUs]	29
incertae sedis	
Calyptella cf. *capula* (Holmsk.) Quél.	3
Calyptella sp.	3
Auriculariales	1
Auriculariaceae	1
Auricularia fuscosuccinea (Mont.) Henn.	1
Auricularia cf. *fuscosuccinea* (Mont.) Henn.	1
Auricularia nigricans (Sw.) Birkebak, Looney & Sánchez-García	1
Auricularia sp.	2
Exidiaceae	
cf. *Exidia* sp.	1
incertae sedis	
Pseudohydnum sp. [2x OTUs]	5
Boletales	
Boletaceae	
Chalciporus sp. Nov.	2
Boletinellaceae	
Boletinellus exiguus (Singer & Digilio) Watling	3
Cantharellales	
Botryobasidiaceae	1
Botryobasidium sp.	1
Clavulinaceae	
Clavulina sp.	1
Multiclavula sp.	1
Geastrales	
Geastraceae	
Geastrum pusillipilosum J.O. Sousa, Alfredo, R.J. Ferreira, M.P Martín & Baseia	1

Geastrum sp. 04 (sensu Zamora et al. 2014)	1
Geastrum cf. *velutinum* Morgan	1
Geastrum sp.	1
Gomphales	1
Gomphaceae	1
Chaetotyphula sp.	1
cf. *Coralloderma* sp.	1
Phaeoclavulina cyanocephala (Berk. & M.A. Curtis) Giachini	1
Ramaria sp. [2x OTUs]	2
Hymenochaetales	
Hymenochaetaceae	5
Coltricia sp.	1
Fuscoporia contigua (Pers.) G. Cunn.	1
Hymenochaete damicornis group (Link) Lév.	2
Hymenochaete cf. *iodina* (Mont.) Baltazar & Gibertoni	3
Hyphodontia sp.	1
cf. *Phellinus* sp.	1
Rickenellaceae	
Peniophorella sp.	1
incertae sedis	
Rigidoporus cf. *microporus* (Sw.) Overeem	1
Rigidoporus sp.	1
Polyporales	3
Hyphodermataceae	1
Incrustoporiaceae	
cf. *Skeletocutis* sp.	1
Irpicaceae	
Irpex rosettiformis C.C. Chen & Sheng H. Wu	2
Meripilaceae	
Rigidoporus (=Physisporinus) umbonatipes group Rajchenb. [3x OTUs]	4
Rigidoporus (=Physisporinus) cf. *vinctus* (Berk.) Ryvarden	2
Cymatoderma caperatum (Berk. & Mont.) D.A. Reid	1
Geesterania cf. *davidii* Westph. & Rajchenb.	2
Merulius tremellosus Schrad.	2
Podoscyphaceae	
Podoscypha sp. [5x OTUs]	7
Polyporaceae [3x OTUs]	7
Amauroderma cf. *praetervisum* (Pat.) Torrend	1
Bresadolia uda (Jungh.) Audet	1
Bresadolia uda complex (Jungh.) Audet	1
Coriolopsis sp.	1
Cubamyces sp. 01	2
Epithele sp. [2x OTUs]	3
Favolus cf. *brasiliensis* (Fr.) Fr.	1
Favolus rugulosus Palacio & R.M. Silveira	1
Favolus sp.	1
Ganoderma australe (clade 2, sensu Morera et al. 2021) (Fr.) Pat.	1

Ganoderma sp.	1
Lamelloporus americanus Ryvarden	1
Lentinus sp.	1
cf. *Microporellus* sp.	1
Neodictyopus gugliottae Palacio, Grassi & Robledo	1
Picipes sp. 01	1
Polyporus guianensis Mont.	7
Polyporus ianthinus Gibertoni & Ryvarden	3
Polyporus tricholoma complex Mont.	2
Tinctoporellus epimiltinus (Berk. & Broome) Ryvarden	1
Trametes membranacea (Sw.) Kreisel	1
Steccherinaceae	
Flabellophora sp.	2
Flaviporus cf. *brownii* (Humb.) Donk	1
Flaviporus liebmanii (Fr.) Ginns	1
Flaviporus sp.	1
cf. *Junghuhnia* sp.	1
Steccherinum undigerum (Berk. & M.A. Curtis) Westph. & Tomšovský	1
Steccherinum sp.	1
Trullella duracina (Pat.) Zmitr.	1
Russulales	
Amylostereaceae	
Artomyces sp.	1
Auriscalpiaceae	
Auriscalpium andinum (Pat.) Ryvarden	1
Auriscalpium sp. [2x OTUs]	2
Lentinellus sp.	1
Lachnocladiaceae	
Lachnocladium sp.	3
Stereaceae	
Stereum sp.	2
Trechisporales	
Hydnodontaceae	
cf. *Fibrodontia* sp.	1
cf. *Lindtneria* sp.	1
Trechispora hondurensis Schoutteten & Haelew.	1
incertae sedis	
incertae sedis	
"*Lactocollybia*" *aurantiaca* Singer	3
Agaricostilbomycetes	
Agaricostilbales	
Chionosphaeraceae	
Chionosphaera phylaciicola (Seifert & Bandoni) R. Kirschner & Oberw	1
Atractiellomycetes	
Atractiellales	
Helicogloeaceae	

Hobsonia mirabilis (Peck) Linder	4
Dacrymycetes	
Dacrymycetales	
Dacrymycetaceae	
Calocera sp.	2
cf. *Calocera* sp.	2
Dacryopinax sp.	1
cf. *Dacryopinax* sp.	1
Pucciniomycetes	
Septobasidiales	
Septobasidiaceae	
Septobasidium sp.	1
Tremellomycetes	
Tremellales	
Tremellaceae	
Tremella sp.	4
**GLOMEROMYCOTA**	
Glomeromycetes	
Diversisporales	
Diversisporaceae	
Redeckera cf. *fulvum* (Berk. & Broome) C. Walker & A. Schüßler	3
Glomerales	
Glomeraceae	
Glomus coremioides (Berk. & Broome) D. Redecker & J.B. Morton	11
